# Plasminogen degrades α-synuclein, Tau and TDP-43 and decreases dopaminergic neurodegeneration in mouse models of Parkinson’s disease

**DOI:** 10.1038/s41598-024-59090-8

**Published:** 2024-04-13

**Authors:** Chunying Guo, Ting Wang, Haiyan Huang, Xiaolu Wang, Yugui Jiang, Jinan Li

**Affiliations:** 1 Department of Applied Research, Talengen Institute of Life Sciences, Room C602G, 289 Digital Peninsula, Shunfeng Industrial Park, No. 2 Red Willow Road, Futian District, Shenzhen, People’s Republic of China; 2 Department of Applied Research, Ruijian Xingze Biomedical Co. Ltd, Dongguan, People’s Republic of China; 3 Department of Basic Research, Talengen Laboratory of Sciences, Shenzhen, People’s Republic of China

**Keywords:** Plasminogen, α-syn, Tau, PD, Dopaminergic neuron, TDP-43, Neuroscience, Neurology

## Abstract

Parkinson's disease (PD) is the second most frequently diagnosed neurodegenerative disease, and it is characterized by the intracellular and extracellular accumulation of α-synuclein (α-syn) and Tau, which are major components of cytosolic protein inclusions called Lewy bodies, in the brain. Currently, there is a lack of effective methods that preventing PD progression. It has been suggested that the plasminogen activation system, which is a major extracellular proteolysis system, is involved in PD pathogenesis. We investigated the functional roles of plasminogen in vitro in an okadaic acid-induced Tau hyperphosphorylation NSC34 cell model, ex vivo using brains from normal controls and methyl-4-phenyl-1,2,3,6-tetrahydropyridine (MPTP)-treated mice, and in vivo in a widely used MPTP-induced PD mouse model and an α-syn overexpression mouse model. The in vitro, ex vivo and in vivo results showed that the administered plasminogen crossed the blood‒brain barrier (BBB), entered cells, and migrated to the nucleus, increased plasmin activity intracellularly, bound to α-syn through lysine binding sites, significantly promoted α-syn, Tau and TDP-43 clearance intracellularly and even intranuclearly in the brain, decreased dopaminergic neurodegeneration and increased the tyrosine hydroxylase levels in the substantia nigra and striatum, and improved motor function in PD mouse models. These findings indicate that plasminogen plays a wide range of pivotal protective roles in PD and therefore may be a promising drug candidate for PD treatment.

## Introduction

Parkinson’s disease (PD) is a progressive neurodegenerative disease characterized by motor symptoms, such as tremors, bradykinesia, and postural instability; nonmotor symptoms, such as cognitive dysfunction, dementia and psychosis; autonomic failure; sleep–wake cycle dysregulation; and depression^1^. PD affects approximately 1% to 2% of adults over age 65 and 4% of adults over age 80^2^. Although PD was first described more than 200 years ago, essentially no effective disease-modifying treatments are currently available. Existing therapies mainly focus on symptom management, and they are often associated with incapacitating side effects^3^. Thus, therapeutic approaches that effectively halt, alleviate or even possibly cure this disease are urgently needed.

One of the typical pathological features of PD is the formation of misfolded protein aggregates (Lewy bodies) of α-synuclein (α-syn), which disrupt factors involved in proteostasis, such as chaperone proteins, the proteasome or autophagy, and eventually lead to the destruction of dopaminergic neurons^4,5^. Current drug development efforts aimed at decreasing α-syn aggregation have focused mainly on using different therapeutic antibodies, but these approaches have not resulted in convincing results of disease improvement^3^. However, accumulating evidence has demonstrated that therapies that target α-syn clearance via proteolytic cleavage or autophagy may provide new therapeutic strategies for PD^4^.

Tau is a microtubule-associated protein, and hyperphosphorylated Tau interacts with α-syn and colocalizes in Lewy bodies to form abnormal inclusion body aggregates in PD^5^. Methyl-4-phenyl-1,2,3,6-tetrahydropyridine (MPTP) is a neurotoxin that specifically induces injury to the nigrostriatal pathway that is accompanied by substantial dopaminergic neurodegeneration. Interestingly, it has been reported that both α-syn and Tau levels are abnormally increased in the MPTP-induced PD mouse model^5^.

The plasminogen activator (PA) system is a general proteolysis system in which the active protease plasmin is formed from its parent protein plasminogen by one of two physiological PAs: tissue-type PAs (tPAs) or urokinase-type PAs (uPAs). Both tPAs and uPAs can be inhibited by the physiological inhibitor-1 (PAI-1), and excessive amounts of plasmin can be inhibited mainly by α2-antiplasmin (α2-AP)^6^.

Several findings from other studies suggest that the PA system is involved in PD. In vitro studies have suggested that plasmin can cleave and degrade α-syn monomers, oligomers and aggregates in a dose- and time-dependent manner^7^. Plasmin can inhibit the translocation of extracellular α-syn to neighboring cells and inhibit the activation of microglia and astrocytes via extracellular α-syn, thereby preventing further α-syn production and propagation^7^. In addition, some studies have shown that the PA system is closely related to pathological processes of nerve degeneration and regeneration after injury, such as remyelination and neuritogenesis^8,9^.

Studies have also suggested that plasmin is involved in activating latent forms of certain neurotrophic factors, such as pro-BDNF (brain-derived neurotrophic factor), to generate their active/mature forms^10^, which play key protective roles in regulating the growth, survival, and differentiation of neurons, including dopaminergic neurons, during PD^11^.

Furthermore, our preliminary studies suggested that administered plasminogen accumulates in the injured area, promoting tissue regeneration, increasing the degradation of misfolded proteins, including α-syn and Tau, and enhancing the repair of nerve injury and dysfunction in both nonneurodegenerative and neurodegenerative diseases^12–16^. In particular, our recent publication showed that during Alzheimer’s disease (AD) development, plasminogen decreases Aβ42 and Tau deposition, increases choline acetyltransferase (ChAT) levels and decreases acetylcholinesterase (AChE) activity, and thus effectively treats AD in mice and humans^17^.

Together, these studies suggest that the PA system may play multiple important roles in neurodegeneration and neuroregeneration in PD. Here, we explored in detail the exact functional roles of plasminogen in PD by in vitro, ex vivo and in vivo experiments. These findings suggest that plasminogen plays a wide range of pivotal protective roles in PD and may be a promising drug candidate for PD treatment.

## Results

### Plasminogen promotes the degradation of α-syn, Ser129-phosphorylated (P-S129) α-syn, hyperphosphorylated Tau and TDP-43 in vitro, ex vivo and in vivo and colocalizes with these proteins

**Figure 1:**
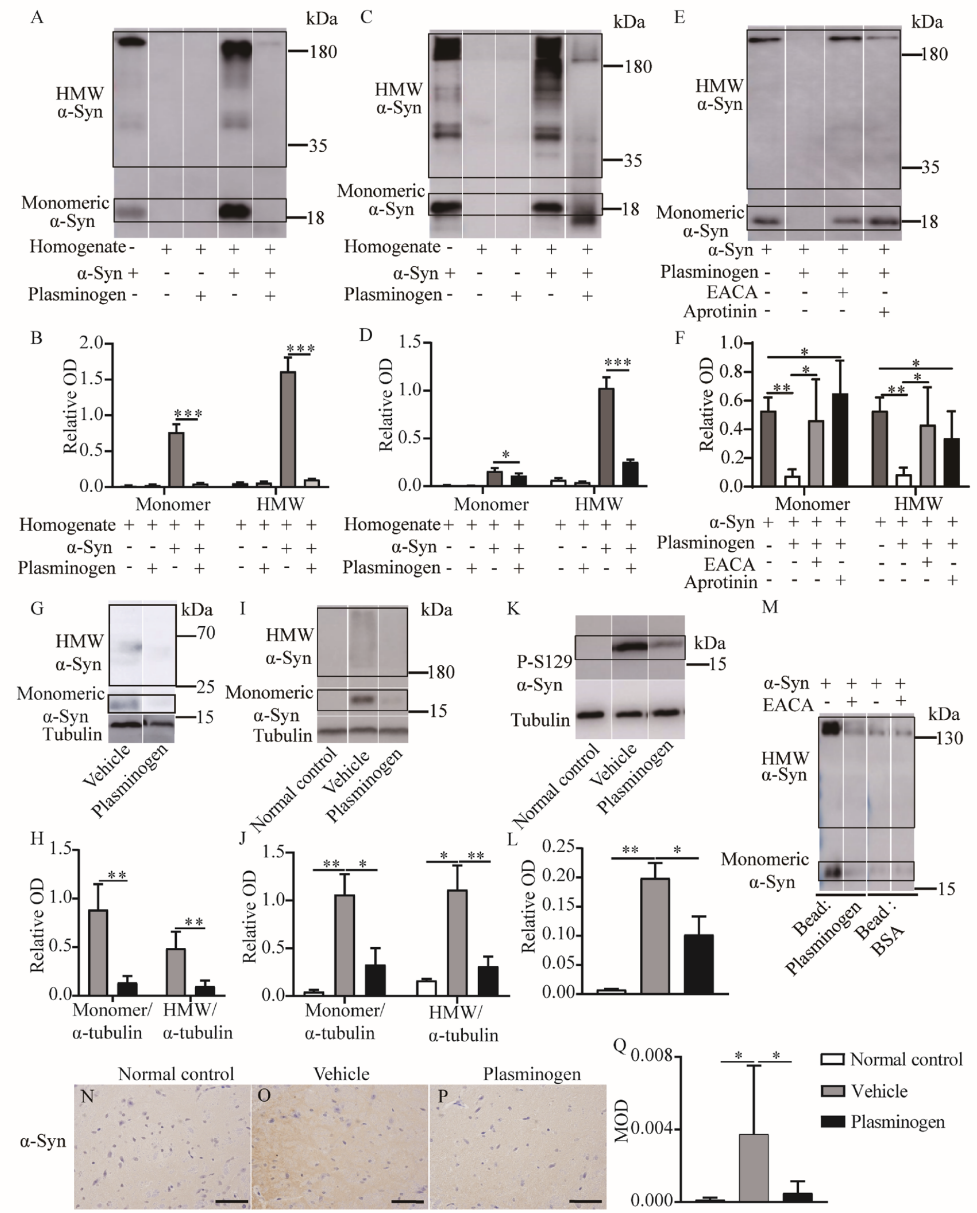
Plasminogen promotes the degradation of α-syn and P-S129 α-syn in vitro, ex vivo and in vivo. (**A**–**D**) Recombinant α-syn protein was mixed with brain homogenates from 5 normal or 5 MPTP-treated C57BL/6 mice and incubated with vehicle or plasminogen for 6 h. (**A**) Representative WB showing α-syn levels in brain homogenates from normal mice after plasminogen incubation; (**B**) Quantitative analysis of the levels of monomeric α-syn (18 kDa) and HMW α-syn (> 33 kDa) in (**A**). (**C**) Representative WB showing α-syn levels in brain homogenates from MPTP-treated mice after plasminogen incubation; (**D**) Quantitative analysis of the levels of monomeric α-syn and HMW α-syn in (**C**). (**E, F**) Recombinant α-syn protein was incubated with saline, plasminogen, plasminogen with EACA or plasminogen with aprotinin for 6 h. (**E**) Representative WB showing α-syn levels after plasminogen incubation; (**F**) Quantitative analysis of the levels of monomeric α-syn and HMW α-syn in (**E**). For **A** to **F**, n = 2 (saline group) or 5 (other groups). (**G**) Representative WB showing α-syn expression in brain homogenates of MPTP-treated mice after plasminogen administration for 14 days; (**H**) Quantitative analysis of the relative levels of monomeric α-syn/α-tubulin and HMW α-syn/α-tubulin in (**G**). n = 7 per group in (**G**, **H**). (**I**) Representative WB showing α-syn expression in brain homogenates of A53T mice after plasminogen administration for 14 days; (**J**) Quantitative analysis of the relative levels of monomeric α-syn/α-tubulin and HMW α-syn/α-tubulin in (**I**). (**K**) Representative WB image of P-S129 α-syn expression in brain homogenates of A53T mice after plasminogen administration for 14 days; (**L**) Quantitative analysis of the relative levels of monomeric P-S129 α-syn /α-tubulin in (**K**). (**I**–**L**) n = 2 in the normal control group, n = 3 in the vehicle-treated group or plasminogen-treated group. (**M**) WB analysis with α-syn antibody (10842–1-AP) of eluates of M-280 Tosylactivated Dynabeads coupled with plasminogen or BSA. (**N**–**P**) Representative images of α-syn immunostaining in the substantia nigra of MPTP-treated mice after plasminogen treatment for 14 days; (**Q**) Quantitative analysis of α-syn immunostaining in (**N**–**P**); n = 5 in the normal control group, n = 8 in the vehicle-treated group, n = 7 in the plasminogen-treated group, scale bar = 50 μm. *P < 0.05; **P < 0.01; ***P < 0.001. OD: optical density. MOD: mean optical density.

To determine whether plasminogen could promote α-syn degradation, we first performed biochemical analysis in an ex vivo or in vitro setting. Extracellular high-molecular-weight (HMW) α-syn was more cytotoxic than monomeric α-syn^18^. Brain homogenates were used to mimic in vivo conditions, because they contain the levels of PAs found in physiological or pathological conditions, particularly tPAs. As shown in Fig. 1A–D, quantitative analysis demonstrated that the levels of both monomeric and HMW α-syn in brain homogenates from both normal and MPTP-treated C57BL/6 mice were significantly lower after incubation with plasminogen than after incubation with the corresponding control agents. The degradation of pure recombinant α-syn by plasminogen was significantly inhibited by aminocaproic acid (EACA), a lysine analog that blocks high-affinity lysine binding sites on plasminogen^19^, or by aprotinin, a direct plasmin inhibitor (Fig. 1E, F).

The in vivo data showed that after 14 days of treatment, the levels of both monomeric and HMW α-syn in the plasminogen-treated group were significantly lower than those in the vehicle-treated group of MPTP-treated mice, or in the α-syn overexpressing A53T mice (Fig. 1G–J). In addition, analysis of the levels of Ser129-phosphorylated (P-S129) α-syn in A53T brains revealed a similar decrease after plasminogen treatment (Fig. 1K, L). These findings were further confirmed by immunohistochemical (IHC) staining for α-syn in the substantia nigra of MPTP-treated mice (Fig. 1N–Q).

Furthermore, an affinity assay showed that magnetic beads coupled to plasminogen precipitated both monomeric and HMW α-syn, and these effects were inhibited by EACA, whereas magnetic beads coupled to bovine serum albumin (BSA) did not precipitate any forms of α-syn (Fig. 1M). This result indicated that plasminogen binds directly to the lysine residues of both forms of α-syn.

**Figure 2:**
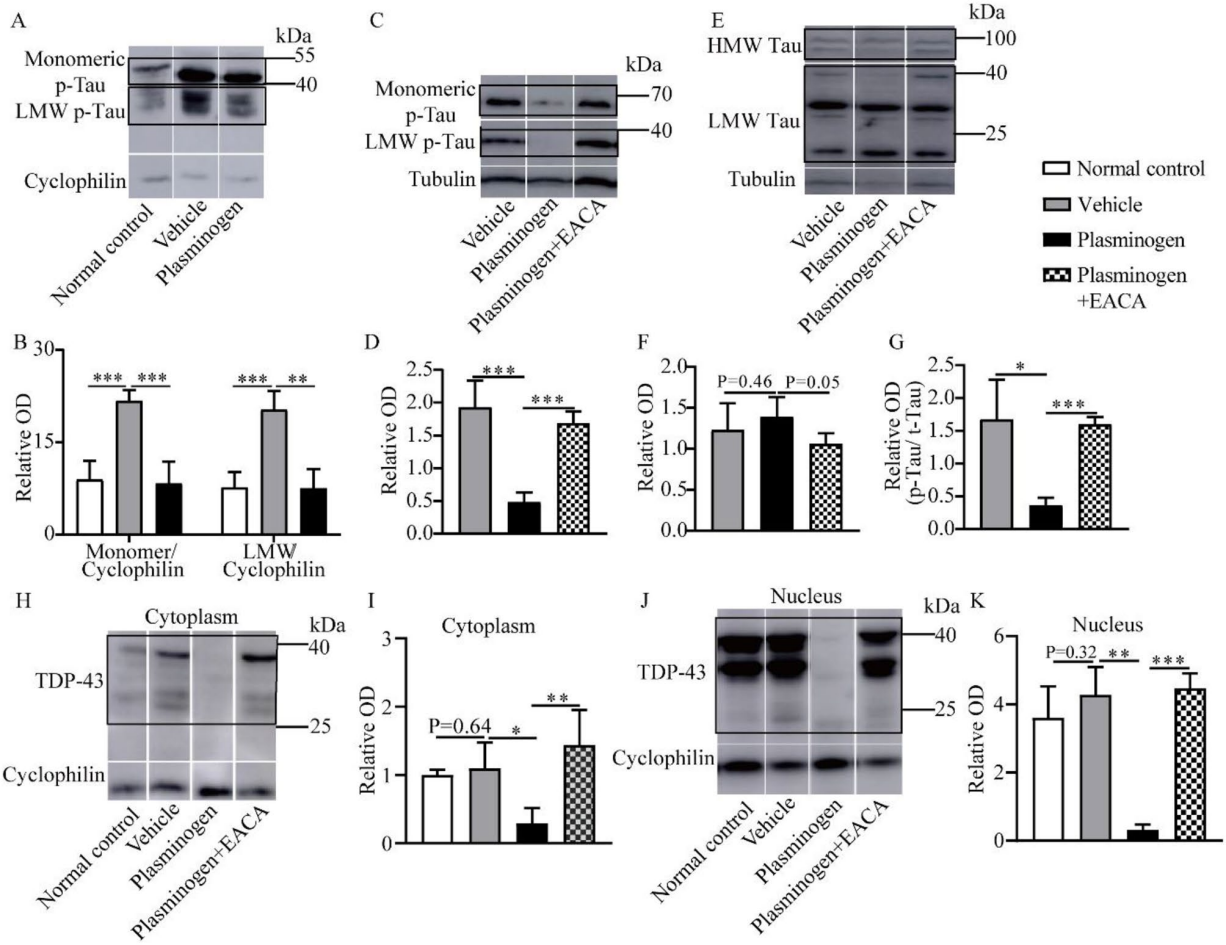
Plasminogen promotes the degradation of hyperphosphorylated Tau ex vivo and in vivo, and of TDP-43 in vitro. (**A**, **B**) WB analysis showing the hyperphosphorylated Tau (p-Tau) protein levels in the brain after the administration of human plasminogen at a dose of 50 mg/kg in MPTP plus LPS-treated mice. (**A**) Representative WB analysis showing p-Tau levels in brain homogenates; (**B**) Quantitative analysis of the relative levels of monomeric (45–65 kDa) and LMW (< 45 kDa) p-Tau in (**A**), n = 5 per group. (**C**–**K**) NSC34 cells were exposed to OA for 24 h and subsequently treated with vehicle, plasminogen or plasminogen with EACA for 24 h. Thereafter, the cells were harvested for WB analysis of the p-Tau and total Tau (t-Tau) protein levels in the whole cells or for determination of the TDP-43 level in the cytoplasm and nucleus. (**C**) Representative WB analysis showing p-Tau levels in OA-pretreated NSC34 cells; (**D**) Quantitative analysis of the relative levels of p-Tau in (**C**). (**E**) Representative WB analysis showing t-Tau levels in OA-pretreated NSC34 cells; (**F**) Quantitative analysis of the relative levels of t-Tau in (**E**); (**G**) Quantitative analysis of the ratio of p-Tau/t-Tau in (**C**, **E**). (**H**) Representative WB analysis showing TDP-43 levels in the cytoplasm of OA-pretreated NSC34 cells that were left untreated (normal control) or treated with vehicle, plasminogen or plasminogen with EACA for 24 h; (**I**) Quantitative analysis of the relative TDP-43 levels shown in (**H**). (**J**) Representative WB analysis showing TDP-43 levels in the nuclei of OA-pretreated NSC34 cells treated with the control, vehicle, plasminogen or plasminogen with EACA treatments for 24 h; (**K**) Quantitative analysis of the relative TDP-43 levels shown in (**J**). n = 5 per group in (**C**–**K**). *P < 0.05; **P < 0.01; ***P < 0.001.

Investigations in the MPTP-induced PD mouse model further showed that in the MPTP plus lipopolysaccharide (LPS)-treated mice, the hyperphosphorylated Tau (p-Tau) protein levels in the brain homogenates significantly increased in the vehicle-treated group, whereas strikingly, 2 h after a single IV injection of plasminogen, both the monomeric and low molecular weight (LMW) p-Tau levels in the plasminogen-treated group were significantly lower than those in the vehicle-treated group (Fig. 2A–B). In addition, compared with those in the corresponding vehicle groups, the total p-Tau antibody reactive band intensities and the p-Tau/total Tau ratio (t-Tau, the intensity of all the bands reactive to the antibody against all the Tau proteins) were significantly lower after plasminogen treatment for 24 h in okadaic acid (OA)-pretreated NSC34 cells, and these effects were significantly inhibited by the addition of EACA (Fig. 2C–E, G). The levels of t-Tau did not change significantly after plasminogen treatment compared to those in the vehicle group (*P* = 0.46; Fig. 2F).

Transactive response DNA-binding protein 43 (TDP-43) is a DNA/RNA binding protein that has been suggested be involved in the copathology of neurodegenerative diseases such as AD and PD^20^. Interestingly, after plasminogen treatment for 24 h, the TDP-43 levels in cytoplasm or nucleus were significantly lower than those in the vehicle-treated group of OA-pretreated NSC34 cells, and these effects were significantly inhibited by EACA (Fig. 2H–K).

Immunofluorescence studies of the striatum and substantia nigra in MPTP-induced PD mice further showed that plasminogen was located both in the extracellular space, in the cytoplasm and in the nucleus of neuronal cells. The immunofluorescence intensity of plasminogen seemed to be negatively correlated with that of α-syn or Tau, and colocalization/adjacency of plasminogen with α-syn or Tau mainly occurred in areas where attenuated α-syn or Tau immunoreactivity was observed (Figs. S1A, B, Fig. S2A, B). This result could be due to the enzymatic degradation effects of plasminogen/plasmin that led to a reduction in the pathological protein α-syn or Tau. In addition, plasminogen also partially colocalized with components of the two intracellular proteolytic systems, LC3 of the autophagy‒lysosome pathway (ALP) and ubiquitin of the ubiquitin‒proteasome system (UPS) (Fig. S1C, D, Fig. S2C, D). Reduced function of the ALP and UPS is a leading cause of neurodegenerative diseases, including PD^21^.

Furthermore, we also found that plasminogen significantly increased the conversion of pro-BDNF to mature BDNF (mBDNF), e.g., by increasing the ratios of mBDNF/pro-BDNF, and thus may contribute to neuroregeneration in the brain. The effect was significantly inhibited by EACA, or by aprotinin (Fig. S3).

### Plasminogen rapidly crosses the blood‒brain barrier (BBB), enters the nucleus and increases plasmin activity in nerve cells

**Figure 3:**
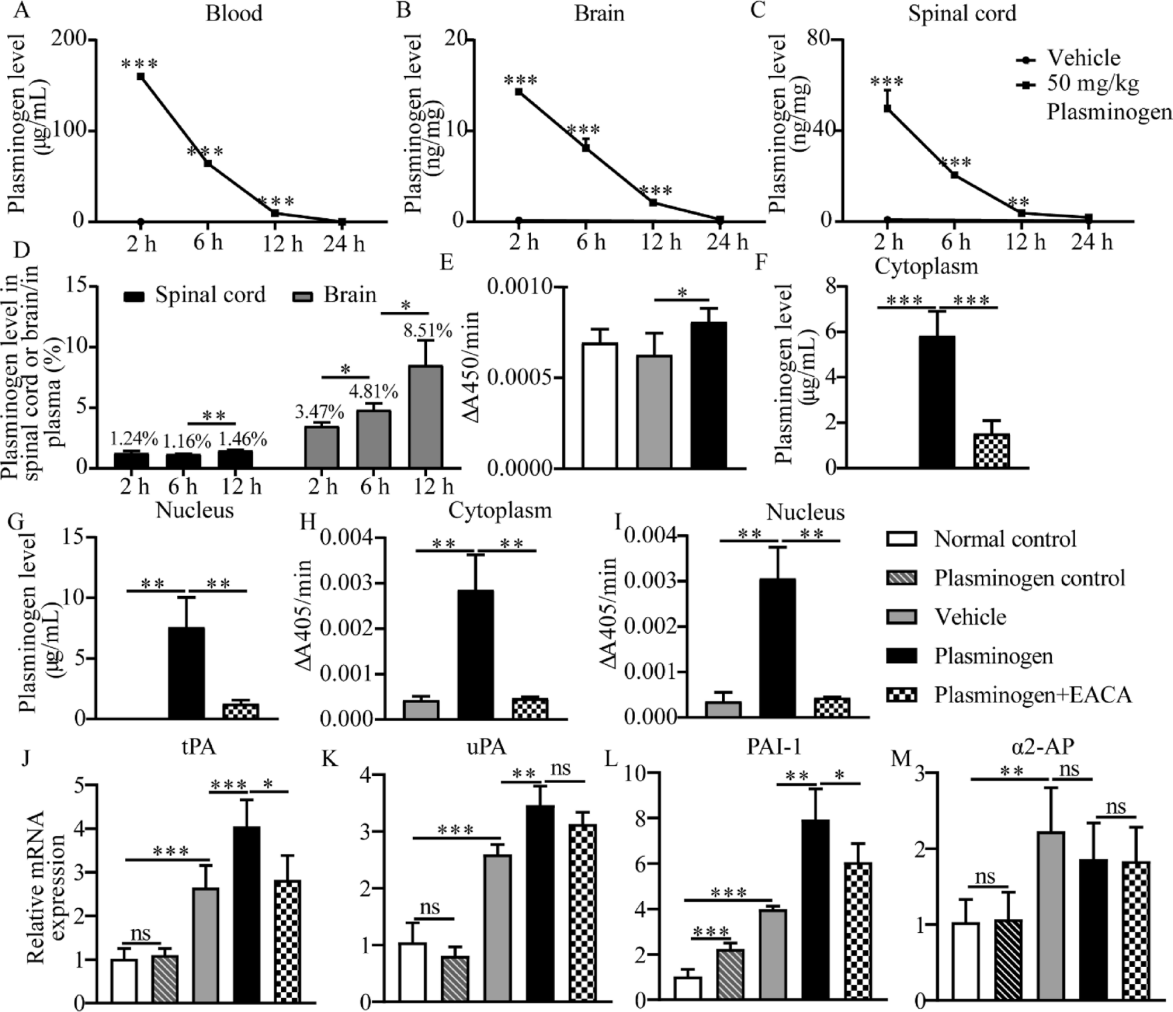
Plasminogen rapidly crosses the BBB and increases plasmin activity in the blood, brain and spinal cord of MPTP-induced PD model mice after which it enters the cytoplasm and nucleus of NSC34 cells. (**A**–**C**) Human plasminogen levels in the blood (**A**), brain (**B**) and spinal cord (**C**) at 2, 6, 12 or 24 h after IV administration of 50 mg/kg human plasminogen. (**D**) The ratio of human plasminogen levels in the spinal cord or brain to that in the blood at 2, 6 and 12 h after IV administration of human plasminogen. (**E**) Plasmin activity in the brain 2 h after the administration of human plasminogen at a dose of 50 mg/kg in MPTP plus LPS-treated mice. n = 3 mice per group in (**A**–**D**); n = 5 mice per group in (**E**). (**F**–**I**) Human plasminogen levels in the cytoplasm (**F**) or nucleus (**G**) and plasmin activity in the cytoplasm (**H**) or nucleus (**I**) of OA-pretreated NSC34 cells after treatment with human plasminogen or human plasminogen with EACA for 24 h. (**J**–**M**) Quantitative comparison of the mRNA levels of the tPA gene (**J**), uPA gene (**K**), PAI-1 gene (**L**), and α2-AP gene (**M**) after human plasminogen or other treatments for 24 h in OA-pretreated NSC34 cells. n = 5 per group in (**F**–**M**). *P < 0.05; **P < 0.01; ***P < 0.001. ns: no significant.

As molecules larger than 400–500 Da usually have difficulty crossing the BBB^22^, we investigated whether the IV-injected macromolecule plasminogen (92 kDa) can cross the BBB under MPTP-induced PD conditions. The quantitative analysis showed that at the 2-, 6-, and 12-h postinjection time points, the levels of human plasminogen in the blood, brain and spinal cord were significantly greater in mice treated with plasminogen than in those treated with vehicle; these levels gradually declined and almost returned to baseline levels after 24 h (Fig. 3A–C).

The average total amount of blood in an adult mouse is approximately 5.85 mL/100 g^23^. Interestingly, during the 12-h postinjection period, although the ratio of the human plasminogen level in the brain at the site of MPTP-induced injury to that in blood significantly increased from 3.47 to 8.51%, the ratio of the human plasminogen level in the spinal cord to that in the blood remained almost constant at approximately 1.2–1.5%. These results suggest that IV-injected human plasminogen specifically accumulated and was maintained for a longer time in the injured area than in the noninjured area of the central nerve system (Fig. 3D).

In addition, the levels of plasmin activity in the brain significantly increased at 2 h after plasminogen administration compared with vehicle administration in MPTP plus LPS-treated PD model mice (Fig. 3E).

Furthermore, in OA-pretreated NSC34 cells, the levels of plasminogen in the cytoplasm and nucleus were significantly greater after human plasminogen treatment for 24 h than in the corresponding vehicle groups, and these effects were significantly inhibited by the addition of EACA (Fig. 3F–G). Correspondingly, the plasmin activity in the cytoplasm and nucleus was significantly increased after human plasminogen treatment and was significantly inhibited by the addition of EACA (Fig. 3H–I).

In addition, the results showed that in OA-pretreated NSC34 cells, the gene expression of tPAs, uPAs and PAI-1 was significantly greater after plasminogen treatment for 24 h than in the vehicle-treated group; moreover, the expression of tPAs and PAI-1, but not uPAs, was significantly inhibited in the presence of EACA (Fig. 3J–L). The expression of α2-AP did not significantly change after human plasminogen treatment (Fig. 3M). These findings showed that plasminogen plays important regulatory roles in the gene expression of members of the plasminogen activation system in NSC34 cells.

### Plasminogen rapidly enters the cytoplasm and further accumulates in the nucleus of NSC34 cells

**Figure 4:**
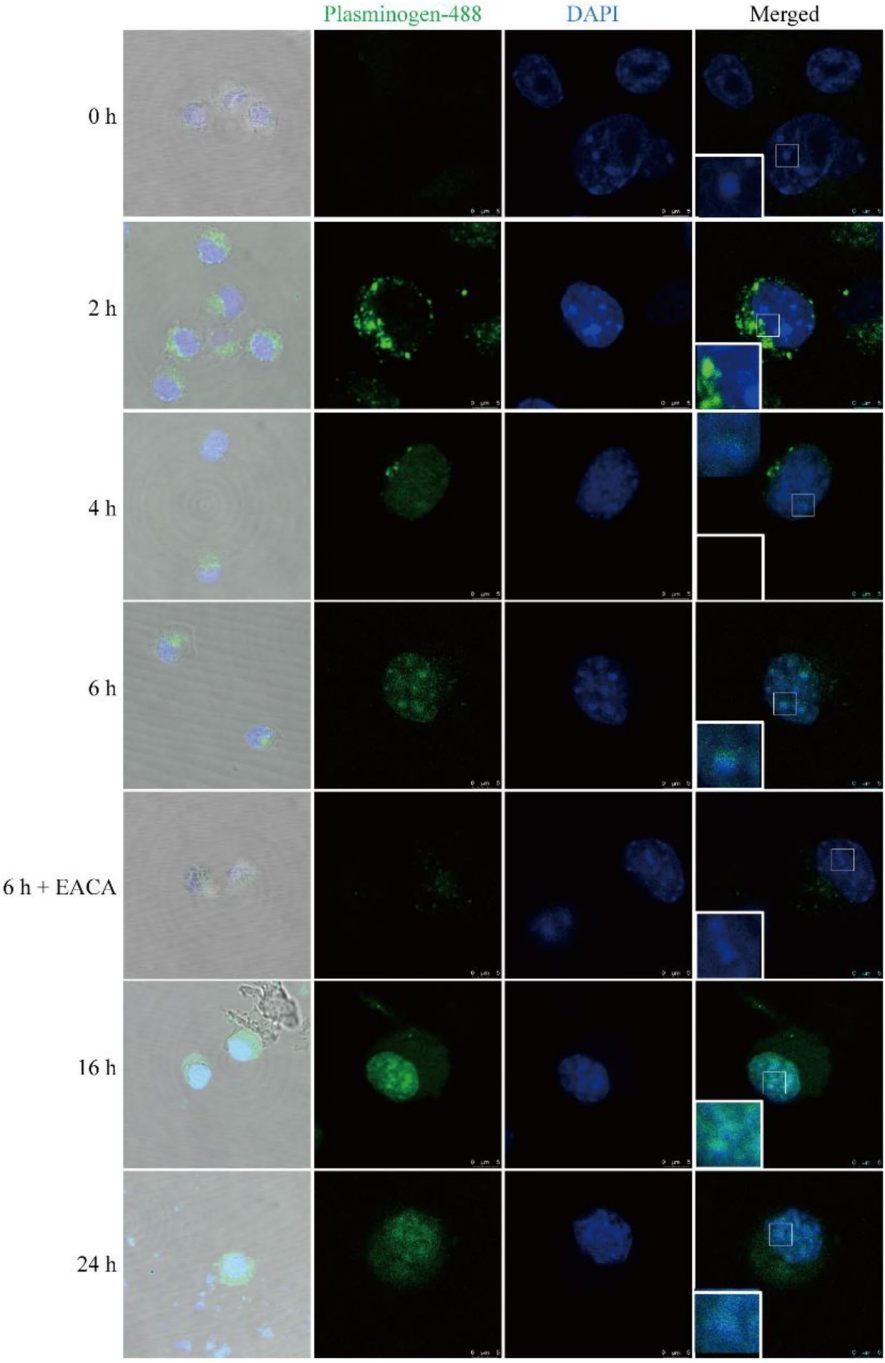
Plasminogen enters the cytoplasm and further accumulates in the nucleus of NSC34 cells. Representative confocal micrograph of OA-pretreated NSC34 cells after administration of plasminogen-488 or plasminogen-488 plus EACA for 0, 2, 4, 6, 16, or 24 h.

By using confocal microscopy, the above findings demonstrating that plasminogen enters the cytoplasm and nucleus were further confirmed by direct observation of the distribution of human plasminogen labeled with the fluorescent dye Alexa Fluor 488 (plasminogen-488) in OA-pretreated NSC34 cells. Within minutes after plasminogen addition (0 h), plasminogen-488 had already begun to enter the NSC34 cells; after 2 h, large amount of plasminogen-488 accumulated on the inner side of the cell membrane and entered the nucleus; after 6 h, most plasminogen-488 had entered the nucleus, but only a few plasminogen-488 molecules entered the nucleus in the additional presence of EACA; after 16 and 24 h, plasminogen-488 further aggregated in the nucleus (Fig. 4). Furthermore, over time, most plasminogen-488 accumulated in the nucleus rather than the cytoplasm (data not shown). These results indicate that plasminogen can quickly enter the cell and aggregate in the nucleus and that lysine binding sites on plasminogen play an important role in these effects.

### Adding plasminogen is neuroprotective and restores normal locomotor activity in MPTP-induced PD model mice

**Figure 5:**
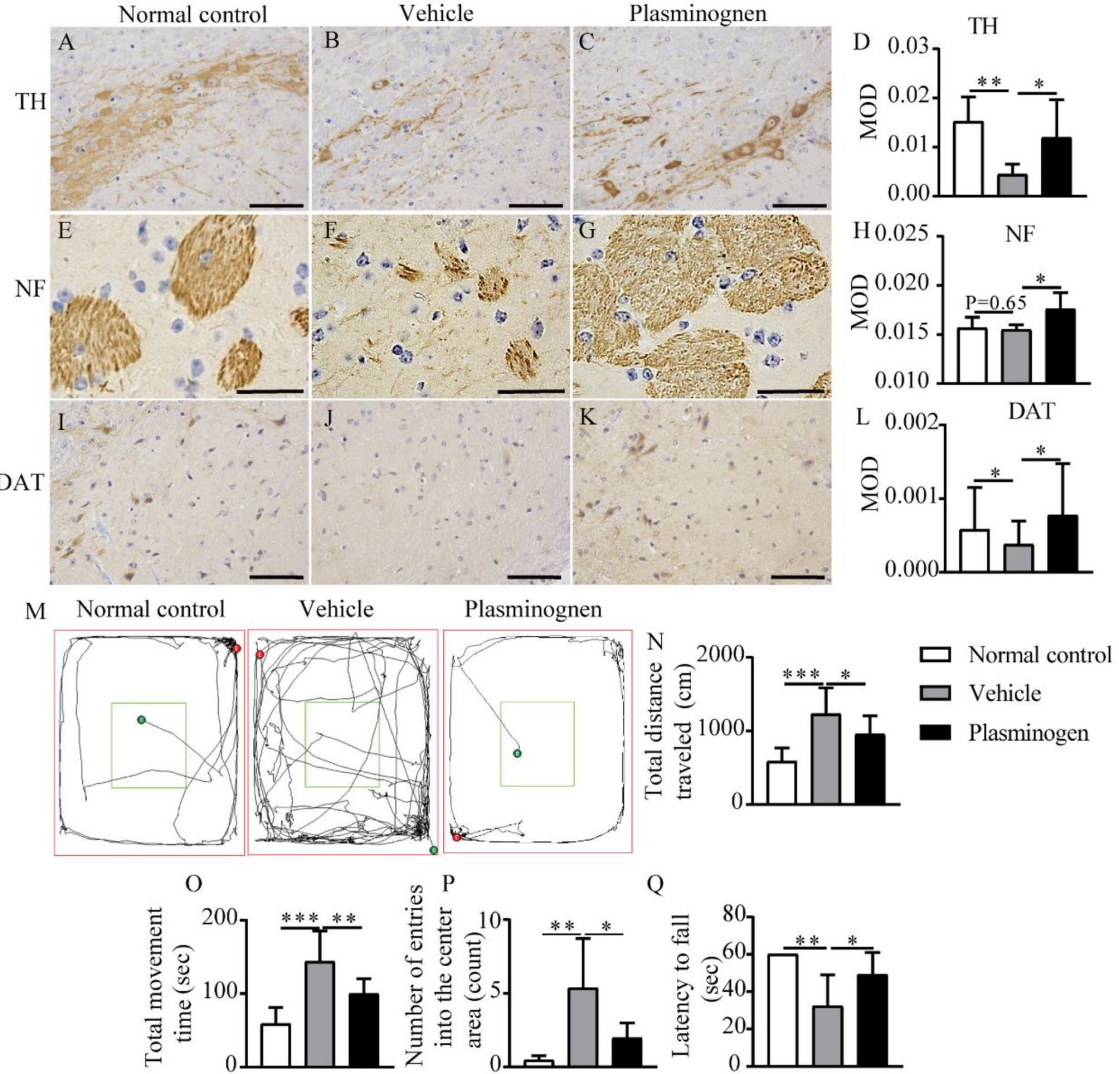
Plasminogen decreases neurodegeneration, promotes neuroregeneration, restores normal locomotor activity in PD model mice. (**A**–**C**) Representative images of TH immunostaining of the substantia nigra; (**D**) Quantitative analysis of the TH immunostaining shown in (**A**–**C**). (**E**–**G**) Representative images of NF immunostaining of the striatum; (**H**) Quantitative analysis of NF immunostaining for (**E**–**G**). (**I**–**K**) Representative images of DAT immunostaining of the substantia nigra; (**L**) Quantitative analysis of the DAT immunostaining data shown in (**I**–**K**). Scale bar = 50 μm. (**A**, **E**, **I**): normal control group (n = 8); (**B**, **F**, **J**): vehicle-treated group (n = 10 in **A**–**H** or 12 in **I**–**L**); (**C**, **G**, **K**) plasminogen-treated group (n = 10 in **A**–**H** or 6 in **I**–**L**). (**M**) Representative images of the travel track in the open field test. (**N**–**P**) Quantitative analysis of the total distance traveled (**N**), total movement time (**O**) and number of entries into the center area (**P**). n = 8 in the normal control group, n = 10 in the vehicle-treated group, and n = 10 in the plasminogen-treated group. (**Q**) Quantitative analysis of the latency to fall in A53T mice in the rotarod test. n = 5 in the normal control group; n = 7 in the vehicle-treated group or plasminogen-treated group. *P < 0.05; **P < 0.01; ***P < 0.001. MOD: mean optical density.

Numerous studies have shown that the accumulation of α-syn likely contributes to the death of dopaminergic neurons, and α-syn clearance might protect against neuronal death^3^. Like in other studies^24^, we found that the abundance of dopaminergic neurons (as indicated by tyrosine hydroxylase (TH) immunostaining intensities) in the substantia nigra was significantly lower in the vehicle-treated MPTP-induced PD model group than in the normal control group, and that this change was abrogated in the plasminogen-treated group (Fig. 5A–D).

In addition, the data showed that the level of neurofilament (NF), which is essential for the structure of normal axons ^25^, in the striatum, and level of dopamine transporter (DAT), which plays a role in dopamine homeostasis by controlling both extracellular and intracellular concentrations of dopamine^26^, in the substantia nigra, were significantly higher in the plasminogen-treated group than in the vehicle-treated group (Fig. 5E–L).

Furthermore, previous studies have reported that treatment of mice with MPTP could induce either locomotion impairment or spontaneous locomotor hyperactivity^27^. In the present study, the results showed that MPTP treatment induced hyperactivity in C57BL/6 mice. Specifically, in the open field test, the total distance traveled, total movement time and number of entries into the center area were significantly greater for MPTP-treated mice than for normal control mice and these changes were abrogated after 14 days of plasminogen treatment (Fig. 5M–P).

Additionally, we found that A53T mice exhibit locomotor impairment. Specifically, in the rotarod test, the latency to fall was significantly lower in the vehicle treated group than in the normal control group, and this decrease was significantly reversed after plasminogen treatment for 7 days (Fig. 5Q).

## Discussion

Numerous clinical studies have demonstrated the presence of both synucleinopathies and tauopathies in PD. Therefore, α-syn and/or Tau clearance has been proposed as a therapeutic strategy for PD for a long time, although limited progress has been made.

As described in the introduction, direct use of plasmin may be important for the development of therapeutic strategies for PD. However, the direct use of plasmin is limited by the fact that plasmin has an extremely short half-life in plasma (< 0.02 s); it is extremely active in blood and is quickly inactivated by α2-AP^28^. On the other hand, plasminogen, which is the inactive preenzyme of plasmin, has a half-life of 19–53 h in plasma and has long been considered relatively inert and abundant in the body, with a plasma concentration of approximately 0.2 mg/mL^6^.

Accordingly, in this study, we found that despite its high molecular weight (over 90 kDa), systemically administered plasminogen rapidly crossed the BBB, enhanced plasmin activity, entered the cells and accumulated in the nucleus, and significantly promoted α-syn, Tau and TDP-43 clearance in vitro, ex vivo and in vivo; we observed similar results in other neurological disease models^17^. Thus, using plasminogen seems to be an attractive alternative strategy to circumvent the potent enzymatic activity and short half-life of plasmin while achieving the goal of effectively degrading α-syn, Tau and TDP-43.

The finding that plasminogen rapidly decreases the levels of hyperphosphorylated Tau within 2 h of plasminogen administration in MPTP-induced PD model mice is surprising. Considering that tauopathy is extensively involved in different neurodegenerative diseases, including PD, AD, frontotemporal lobar degeneration and Pick’s disease, these findings further suggest the therapeutic potential of plasminogen in these diseases.

Furthermore, it is widely accepted that plasmin/plasminogen exerts cell-associated activities by binding to the cell surface via surface plasminogen receptors. The binding of plasminogen with these cell receptors triggers downstream intracellular signaling pathways associated with immune cell recruitment, inflammation modulation and improved wound healing^29^. To date, at least twelve plasminogen receptors have been identified, and their functions have been extensively studied^29^. However, by using confocal microscopy, ELISA and plasmin activity analysis, we demonstrated that plasminogen itself rapidly entered the cytoplasm and that most plasminogen eventually accumulated in the nucleus. Correspondingly, we also observed that cytoplasmic and nuclear TDP-43 levels were significantly decreased by the addition of plasminogen, and these effects were abrogated after the lysine analog EACA was added. These findings clearly demonstrated that plasminogen itself directly plays intracellular and intranuclear roles, and it seems that such roles are effective and specific in degrading pathogenic proteins such as TDP-43 and Tau. These findings help explain our other observations and further open the possibility that treatment with plasminogen may play direct important regulatory roles intracellularly and intranuclearly by degrading other known or unknown toxic/pathogenic proteins, interacting with other subcellular compartments such as the ALP and UPS, as shown here; and regulating gene expression patterns, for instance genes members of the plasminogen activation system. Therefore, the addition of plasminogen may directly play multidimensional protective roles intracellularly and intranuclearly, leading to comprehensive healing effects on the body.

**Figure 6:**
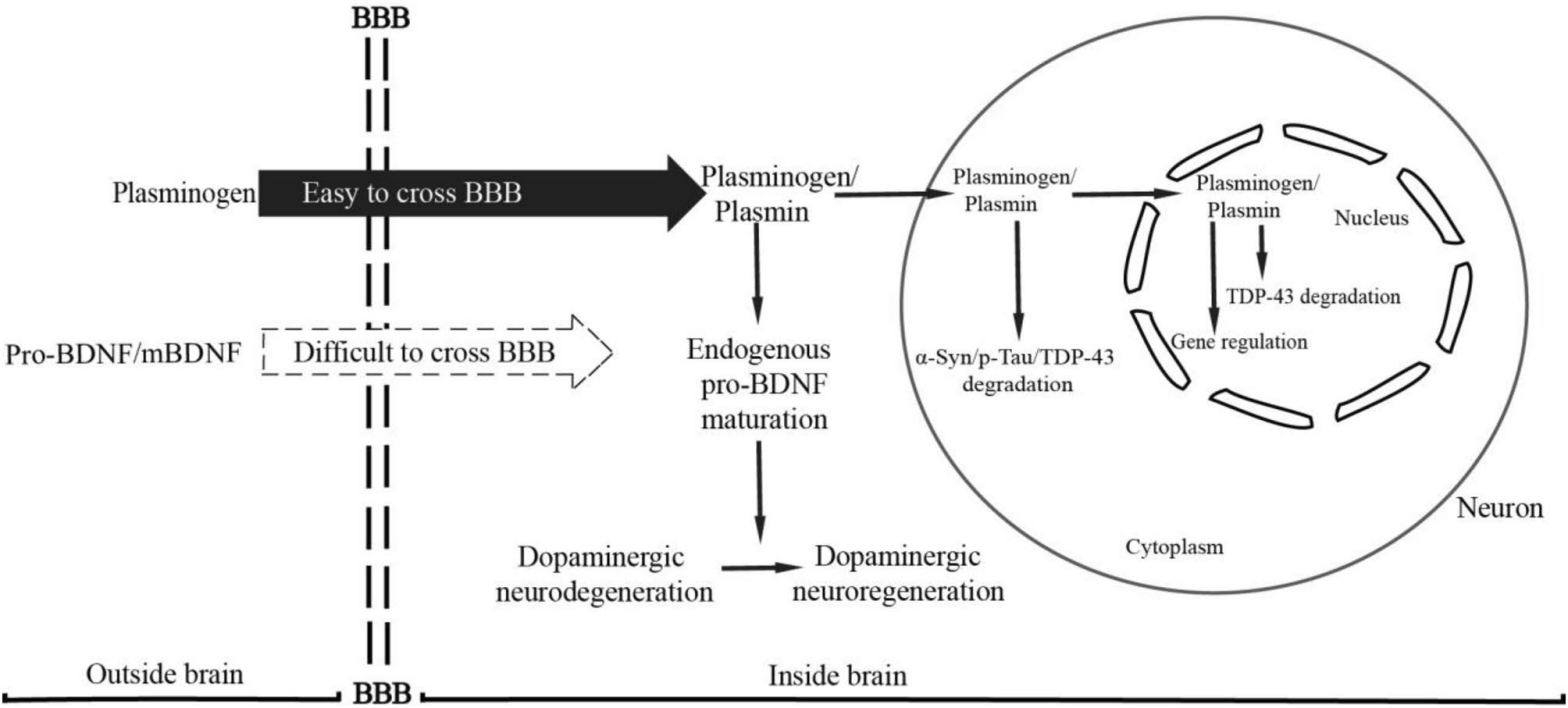
Schematic illustration of the possible mechanisms by which plasminogen treats PD. Administered plasminogen rapidly crosses the BBB to reach areas with α-syn or Tau aggregates/sites associated with PD pathogenesis; these areas are also where PAs, particularly tPAs, are locally expressed at high levels and further convert plasminogen to active plasmin. By binding to the lysine residues on these proteins via lysine binding sites, plasminogen/plasmin quickly decreases the aggregation of intercellular pathogenic proteins, including α-syn and Tau, fibrin, and possibly other unidentified conformationally abnormal or denatured proteins, which also help decrease further intercellular propagation of these proteins and related disease progression. In addition, through high lysine-binding affinities, plasminogen/plasmin rapidly enters the cytoplasm of neurons, possibly by endocytosis (reference^30^ and data from the current study).Moreover, plasminogen/plasmin binds to intracellular pathogenic proteins, such as α-syn, TDP-43, and Tau aggregates by recognizing the exposed lysine residues on these proteins and directly clears them via proteolytic degradation or indirectly clears them by colocalizing and possibly interacting with the intracellular UPS or ALP to further enhance their protein degradation functions. In addition, through high lysine-binding affinities, most plasminogen/plasmin gradually accumulates in the nucleus of neurons/cells, where plasminogen/plasmin degrades intranuclear pathogenic proteins such as TDP-43, as observed in the present study, and exerts gene regulatory functions, such as effects on members of the plasminogen activation system. In addition, through high lysine-binding affinities, plasminogen/plasmin also increases the abundance of mature neurotrophic molecules, such as mBDNF, by promoting the activation and maturation of pro-BDNF to exert its neuroprotective effect on damaged dopaminergic neurons, as indicated by the increased expression of DAT, TH and NF in the present study. Together, owing to its strong lysine binding properties and potent enzymatic properties, the application of plasminogen seems to simultaneously promote the ability of the PA system to promote neuroregeneration and protect against neurodegeneration, both at the molecular level, subcellular level and cellular level, eventually leading to improvements in PD pathology.

Therefore, as shown in Fig. 6, one possible molecular mechanism by which plasminogen functions in treating PD is proposed. This hypothesis provides a theoretical framework/therapeutic strategy for treating this devastating neurodegenerative disease, although substantial detailed investigations and additional evidence are needed to further explore this hypothesis.

Disrupted regulation of dopamine/DAT/dopaminergic neurons has also been suggested to be related to other psychiatric disorders, such as schizophrenia, autism spectrum disorders, addictive disorders, attention-deficit/hyperactivity disorder (ADHD), autism spectrum disorders, bipolar disorder and depression disorders^26,31^. In the present study, we observed that the levels of TH, NF, and DAT, which are key markers of dopaminergic neurons and axon growth, significantly increased in the substantia nigra or striatum after plasminogen treatment^24,25^. The ex vivo study also showed that plasminogen promoted the transformation of pro-BDNF to mBDNF, which is known to be key for enhancing the survival of dopaminergic neurons. Thus, these observations of the treatment effects of plasminogen on dopaminergic neurons may provide evidence of the therapeutic potential of plasminogen for the abovementioned psychiatric disorders. Indeed, for instance, after plasminogen treatment, our early preliminary results have shown psychiatric improvements in the cuprizone (CPZ) model, which is a widely used animal model of schizophrenia^32^, and in patients with neurological diseases who exhibit psychiatric symptoms^33^. Undoubtedly, this is a large and complex series of mechanisms, and further investigations of the molecular mechanisms underlying these observations are needed.

The spinal cord plasminogen levels in the present study on PD model mice were significantly different from those in our previously published study on AD model mice^17^. This difference is probably due to the different mouse strains used, the different disease processes involved, and therefore, the differences in insult levels and host response patterns between the two studies. In this study, the mouse strains used were normal C57BL/6 and B6/JGpt-Tg(hSNCA-A53T)62/Gpt transgenic mice, whereas in the AD study, B6SJL-Tg(APPSwFlLon,PSEN1*M146L*L286V)6799Vas/Mmjax transgenic mice were used.

Plasminogen has been approved for threating rare type I plasminogen deficiency disease in human patients. In the clinical trial, all patients achieved at least an absolute 10% increase in trough plasminogen activity above baseline after plasminogen therapy. Plasminogen was well tolerated in both children and adults and there were no deaths or serious adverse events^34^. Similar to the clinical data on plasminogen deficiency, our preclinical data showed that the levels of human plasminogen in the blood, brain and spinal cord were significantly increased in PD model mice after plasminogen administration (Fig. 3). These findings suggest that, regardless endogenous plasminogen level, plasminogen supplementation may increase the plasminogen level and thus affect the homeostasis of plasminogen in vivo.

In conclusion, this is the first study to investigate the functional roles and molecular mechanism of plasminogen in treating PD in mice. Although the findings are interesting, especially considering the poor treatment choices that are currently available and the devastating nature of this disease, additional preclinical and clinical studies are needed to fully understand the molecular mechanisms and clinical efficacy of plasminogen in PD treatment. Such work is ongoing at our institute.

## Materials and methods

### Animals and reagents

All mice were housed in a humidity- and temperature-controlled environment maintained on a 12:12-h light:dark cycle and were fed standard rodent chow and water. The experiments were performed on 10- to 24-week-old male littermates of the C57BL/6 genetic strain or 7- to 8-week-old B6/JGpt-Tg(hSNCA-A53T)62/Gpt (A53T) genetic strain. A53T mice overexpress α-syn with the A53T mutation. When the mice were approximately 8 weeks of age, they developed α-syn aggregation, dopaminergic neurodegeneration, an increase in Ser129-phosphorylated (P-S129) α-syn, a decrease in motor coordination and limb strength, a decrease in body weight and a decrease in survival; these conditions are all typical phenotypes of PD (Gempharmatech Co., Ltd, P.R.China). C57BL/6 mice were purchased from Guangdong Medical Laboratory Animal Center, P.R.China. A53T mice (stock number T054329) and A53T wild-type (background: C57BL/6JGpt) mice were purchased from Gempharmatech Co., Ltd, P.R.China. The main reagents and antibodies used in the present study are listed in Table S1. All the experimental procedures were approved by the Institutional Animal Care and Use Committee of the Talengen Institute of Life Sciences, Shenzhen, China. All the experiments were performed in accordance with the recommendations in the Guide for the Care and Use of Laboratory Animals of National Administration Regulations on Laboratory Animals of China and were reported as described by ARRIVE guidelines. All the mice used in the present study were euthanized by excessive inhalation of isoflurane.

### Establishment of the MPTP-induced PD mouse model

The mice were injected intraperitoneally with MPTP dissolved in saline (35 mg/kg) once per day for a total of 5 days to establish the PD model^35^. After the model was established, all the mice were housed individually and administrated vehicle or plasminogen for the indicated times.

In some studies, 5 mg/kg LPS was administered by intraperitoneal injection 24 h after the final MPTP injection (referred to MPTP plus LPS) to induce p-Tau expression in the vehicle-treated group and the plasminogen-treated group, and 24 h thereafter, a single dose of vehicle or plasminogen was administered by IV injection.

### Human plasminogen administration

The MPTP-treated mice or A53T mice were divided into 2 groups: the vehicle-treated group, and the plasminogen-treated group. C57BL/6 or A53T wild type mice were used as normal controls. In the plasminogen-treated group, 50 mg/kg human plasminogen in 5 mL/kg vehicle was intravenously (IV) injected daily. The mice in the vehicle-treated group were IV injected with 5 mL/kg vehicle (10 mM citric acid sodium citrate solution, pH 7.4) daily. The mice in the normal control group were treated with saline or not treated with any other agents. During the experiments, the mice were subjected to the open field test or rotarod test to assess their motor functions. At the end of the experiment, tissue samples were harvested for immunohistochemical analysis, enzyme-linked immunosorbent assay (ELISA) or western blot (WB) analysis.

### Ex vivo experiments

Ex vivo experiments were performed by incubating plasminogen together with recombinant human α-syn or human Pro-BDNF in combination with saline or brain homogenates from the PD model and normal C57BL/6 mice. Briefly, brain homogenates were generated in PBS from the brain tissues of 5 mice with MPTP-induced PD or 5 normal C57BL/6 mice, and these samples were mixed and centrifuged (12,000 rpm, 20 min, 4 °C) before use. Then, 21.5 μg of recombinant α-syn or 21.5 μg of Pro-BDNF was added to the brain homogenates or saline and incubated with 9.2 μg of plasminogen, vehicle (for α-syn studies, 10 mM citric acid sodium citrate solution, pH 7.4), plasminogen with aminocaproic acid (EACA) (478 mM) or plasminogen with aprotinin (146 μM) in a total reaction volume of 50 μL at 37 °C for 6 h in vitro. The degradation of α-syn or Pro-BDNF was then stopped by the addition of 0.1% trifluoroacetic acid solution buffer. Thereafter, the samples were analyzed by WB.

### Okadaic acid (OA) induced Tau hyperphosphorylation in NSC34 cells and plasminogen treatment

NSC34 cells were seeded in 9 cm^2^ cell culture plates at a density of 10^6^ cells/well. NSC34 cells were cultured in DMEM supplemented with 10% FBS in an incubator at 37 °C with high humidity and 5% CO_2_. Two days after seeding, the NSC34 cells were exposed to OA at 2.5 ng/μL. After OA exposure for 24 h, the cells were grouped as follows: vehicle group: the cells were treated with vehicle; plasminogen-treated group: the cells were treated with plasminogen at 0.5 mg/mL; and plasminogen with EACA group: the cells were treated with plasminogen at 0.5 mg/mL together with 20 mM EACA. Once vehicle, plasminogen or plasminogen with EACA was added, the medium was not changed until total protein was subsequently isolated. After plasminogen treatment for 24 h, the cells were harvested for ELISA, WB analysis, plasmin activity analysis, or reverse-transcription polymerase chain reaction (RT‒PCR).

### Extraction of cytoplasmic and nuclear proteins

The cultured cells were digested with 2.5 g/L trypsin for 2–3 min, and the digestion was terminated with DMEM. Then, the cells were centrifuged (1500 rpm, 4 °C, 5 min), and the supernatant was removed. Cytoplasmic and nuclear proteins were extracted according to the operation manual of the nuclear protein extraction kit. In brief, the cells were added to plasma protein extraction reagents (200 μL/20 μL cells), vortexed at high speed for 15 s, incubated on ice for 10 min, vortexed again at high speed for 10 s and centrifuged at 13,400×*g* and 4 °C for 10 min. The supernatant was the extracted cytoplasmic protein, and the precipitate contained the extracted nuclear protein. The precipitate was further mixed with nuclear protein extraction reagents, vortexed at high speed for 15 s, incubated on ice for 10 min, vortexed again at high speed for 10 s and centrifuged at 13,400×*g* and 4 °C for 10 min. The supernatant was subsequently obtained, which was the extracted nuclear protein. The extracted cytoplasmic and nuclear proteins were subjected to human plasminogen ELISA and plasmin activity detection.

### Adherence of OA-pretreated NSC34 cells to coverslips and plasminogen-488 treatment

Predisinfected coverslips were first placed in 24-well plates. NSC34 cells were seeded at 5 × 10^4^ cells/well density. NSC34 cells were cultured in DMEM supplemented with 10% FBS and a penicillin/streptomycin cocktail (100 UI/mL and 100 μg/mL, respectively) in an incubator at 37 °C with high humidity and 5% CO_2_. Two days after seeding, the NSC34 cells were exposed to OA at 2.5 ng/μL. After OA exposure for 24 h, the cells were treated with Alexa Fluor 488-labeled human plasminogen (Plasminogen-488) or plasminogen-488 plus EACA. The coverslips were removed after 0, 2, 4, 6, 16 or 24 h and fixed with 4% paraformaldehyde solution. Then, the cells were analyzed by immunofluorescence and confocal laser scanning microscopy. Plasminogen was labeled with Alexa Fluor 488 dye by KMD Bioscience using the coupling method.

### Confocal scanning laser microscopy

Coverslip-adherent OA-pretreated NSC34 cells were fixed with 4% paraformaldehyde for 10 min, stained with DAPI for 5 min, and sealed with glycerogelatin. The signals were captured with a confocal scanning laser microscope (Leica TCS SP8, Germany).

### Reverse-transcription polymerase chain reaction (RT‒PCR)

Total RNA was extracted from tissues using an RNA Easy Fast Kit. The total RNA quality and quantity were evaluated for each RNA sample using a microplate spectrophotometer (Epoch, BioTek, US). One hundred nanograms of total RNA was reverse-transcribed using the Abstart One Step RT‒PCR Mix Kit, and tPA regions, uPA regions, α2-antiplasmin regions, and PAI-1 regions were PCR-amplified using the primers shown in Table S2. Tubulin was used as an internal control.

### Open field test

The analysis of locomotion in the open field test is the most common procedure for measuring motor function and is one of the oldest tests used for assessing rodents. The test was performed essentially as described previously^27^.

### Rotarod test

For the rotarod test, the time for which an animal could remain on the rotating cylinder (3.5 cm) of a rotarod apparatus (Chengdu Techman Software Co., Ltd, 213-200, P.R.China) at a constant speed of 16 rpm was measured. Each animal was tested in three trials, and the longest latency to fall was recorded; 180 s was chosen as the arbitrary cutoff time.

### Affinity assay with magnetic beads

The affinity assay was performed essentially according to the user manual for Dynabeads M-280 Tosylactivated. The ligands used were plasminogen or bovine serum albumin (BSA). Then, 100 μg (165 μL) of ligand and 5 mg (165 μL) of M-280 Tosylactivated Dynabeads were incubated on a roller at room temperature for 22 h, and then placed in a magnet for 2 min to obtain the precipitate. The mixture was washed with 0.1% BSA 2 times, incubated with 3% BSA on a roller at 37 °C for 4 h for blocking, washed with 0.1% BSA 3 times, and placed on a magnet for 2 min to obtain the magnetic beads coupled to the ligand, which were stored in 0.1% BSA with a volume of 240 μL at 4 °C until further use.

Ten micrograms of recombinant human α-syn alone or 10 μg of recombinant human α-syn premixed with 14.8 mM EACA was added to 50 μL of magnetic beads coupled to plasminogen or BSA, incubated on a roller at room temperature for 1 h, and placed on a magnet for 2 min to precipitate. The mixture was further washed with 0.5% BSA 3 times, and placed on the magnet again for 2 min to obtain the final precipitate.

Thereafter, the precipitate was mixed with 20 μL of eluent (0.2 M glycine, pH 2.5), incubated at room temperature for 10 min, and placed on a magnet for 2 min. The supernatant was collected and added to a new tube containing 2 μL of neutralizing buffer (2 M Tris), after which the pH was adjusted to neutral. Thereafter, the four groups of samples were subjected to WB analysis for α-syn.

### WB analysis

The analysis was performed as described in previous studies^17^. Anti-α-syn (1:2000), anti-P-S129α-syn (1:1000), anti-Tau (1:2000), anti-TDP-43 (1:1500) and anti-BDNF (1:4000) antibodies were used in the WB analysis. Detection and quantification of band optical density (OD) were conducted using ImageJ analysis software. All bands at the correct molecular weight were analyzed as the signal for that target protein. The ex vivo and in vitro data were normalized by dividing the OD value of the target protein in the lanes containing the samples incubated with plasminogen, vehicle, plasminogen with EACA or plasminogen with aprotinin by the OD value of the target protein in the lanes containing the samples incubated with saline alone within the same gel. The data from the cell and in vivo experiments were normalized to the loading control protein (the so-called relative OD) by dividing the OD value of the target protein by the OD value of the loading control in the same gel.

### Immunohistochemical (IHC) analysis

The analysis was performed as described in previous studies^17^. Anti-α-syn (1:1200), anti-TH (1:400), anti-NF (1:4000) and anti-DAT (1:600) primary antibodies were used for IHC analysis. IPP6.0 pathological image analysis software was used to determine the integral optical density (IOD) of immunopositive area of interest. The IOD is the sum of the individual pixels within the field of view of the specimen. The mean optical density (MOD) is the total value of pixels in the area of interest (IOD SUM) divided by the area to be tested. MOD represents the intensity of protein expression.

### Other methods

The metabolism and distribution of plasminogen and in vivo human plasminogen antigen levels were measured via ELISA. Additionally, plasmin activity was assessed with the substrate S-2251, and immunofluorescence analysis was performed as described in previous studies^17^. Anti-plasminogen (1:800), anti-α-syn (1:1200), anti-Tau (1:1000), anti-ubiquitin (1:1600) and anti-LC3B (1:200) antibodies were used for immunofluorescence analysis.

### Statistical analysis

The data are presented as the mean ± SD. Mean differences between the groups were analyzed using one-way ANOVA with SPSS 16.0. A *P* value < 0.05 was considered to indicate statistical significance.

### Ethics statement

All the experimental procedures were approved by the Institutional Animal Care and Use Committee of the Talengen Institute of Life Sciences, Shenzhen, China.

### Supplementary Information

Supplementary Information 1.

Supplementary Information 2.

## Data Availability

The datasets used and/or analyzed during the current study are available from the corresponding author upon reasonable request.
