# Supplementary material for: Plasminogen degrades α-synuclein, Tau and TDP-43 and decreases dopaminergic neurodegeneration in mouse models of Parkinson’s disease
Source: Sci Rep. 2024 Apr 13;14:8581. doi: 10.1038/s41598-024-59090-8 (PMC11016066; doi:10.1038/s41598-024-59090-8)
Supplement: Supplementary file 1 — Supplementary Information 1. [file 41598_2024_59090_MOESM1_ESM.pptx]

## Slide 1
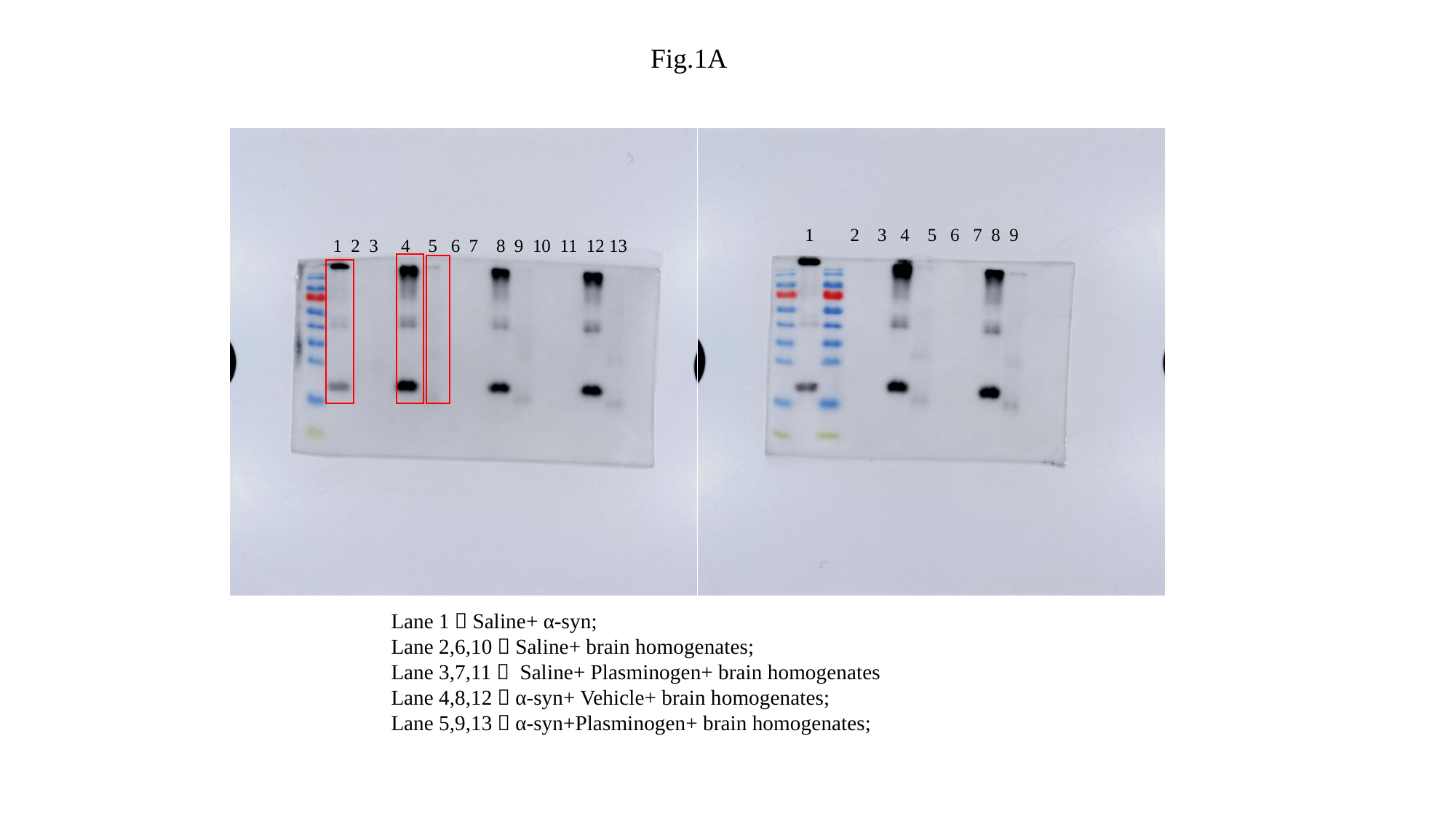

Fig.1A
1 2 3 4 5 6 7 8 9
1 2 3 4 5 6 7 8 9 10 11 12 13
Lane 1：Saline+ α-syn;
Lane 2,6,10：Saline+ brain homogenates;
Lane 3,7,11： Saline+ Plasminogen+ brain homogenates
Lane 4,8,12：α-syn+ Vehicle+ brain homogenates;
Lane 5,9,13：α-syn+Plasminogen+ brain homogenates;

## Slide 2
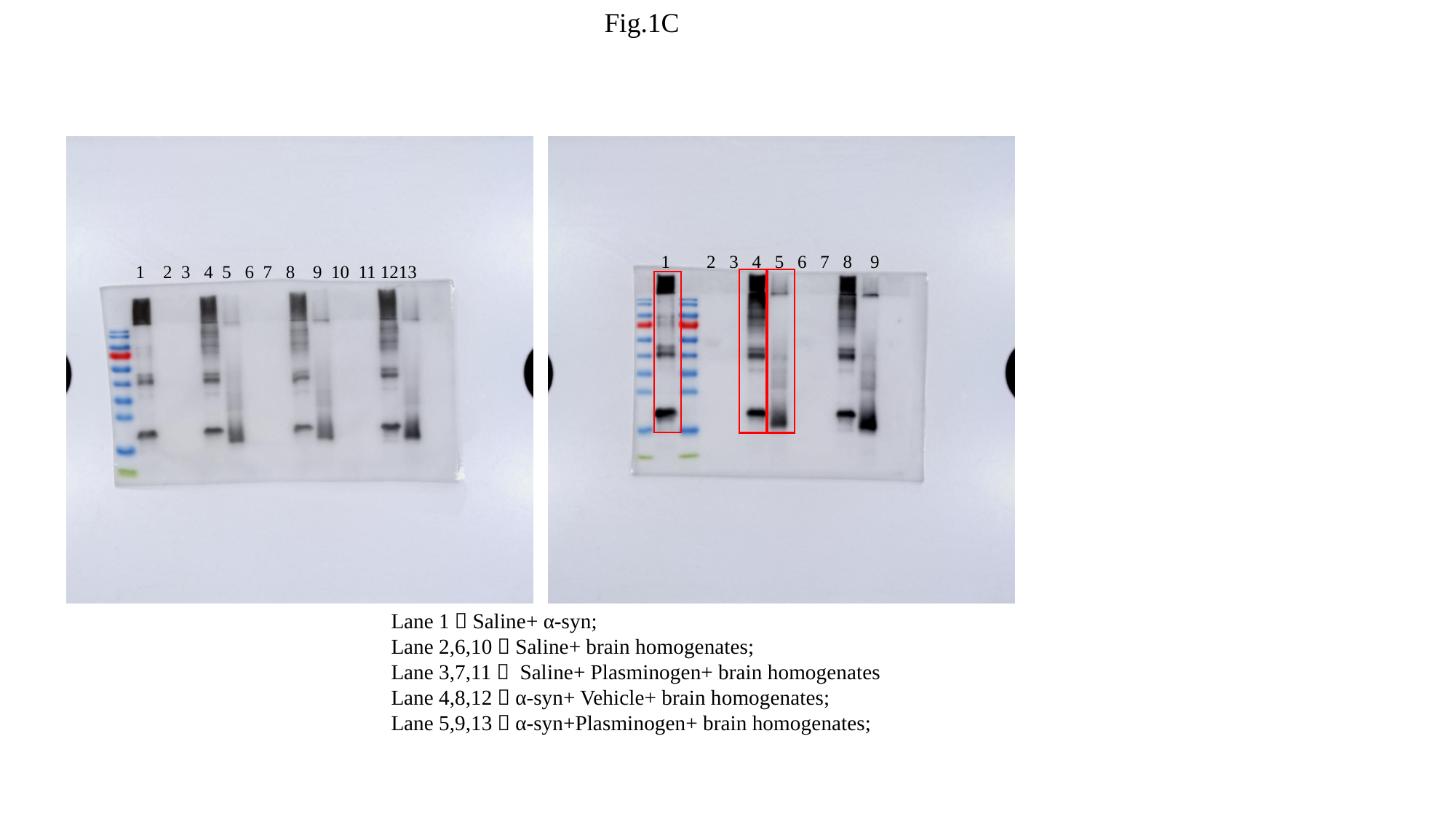

Fig.1C
1 2 3 4 5 6 7 8 9
1 2 3 4 5 6 7 8 9 10 11 1213
Lane 1：Saline+ α-syn;
Lane 2,6,10：Saline+ brain homogenates;
Lane 3,7,11： Saline+ Plasminogen+ brain homogenates
Lane 4,8,12：α-syn+ Vehicle+ brain homogenates;
Lane 5,9,13：α-syn+Plasminogen+ brain homogenates;

## Slide 3
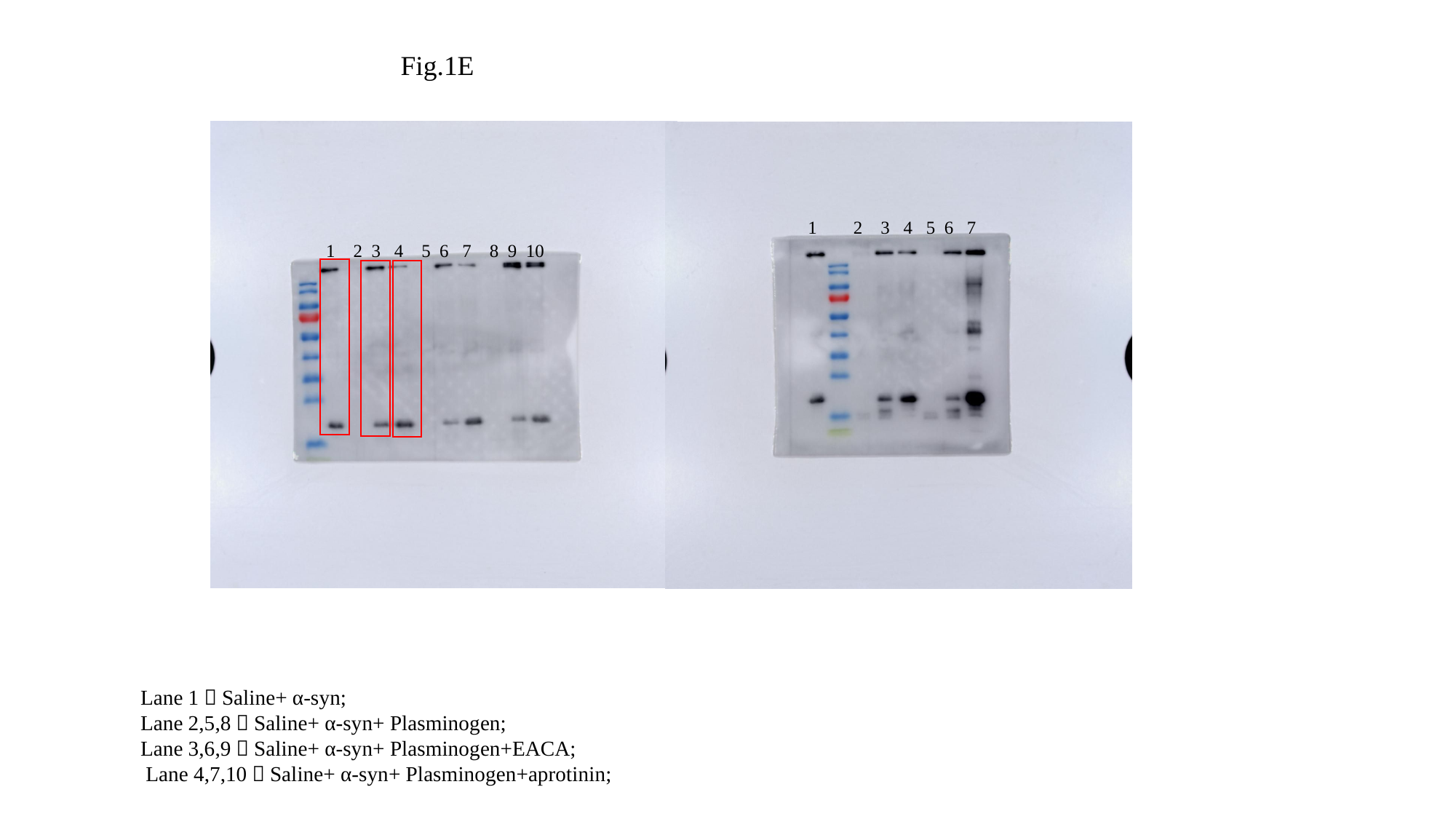

Fig.1E
1 2 3 4 5 6 7
1 2 3 4 5 6 7 8 9 10
Lane 1：Saline+ α-syn;
Lane 2,5,8：Saline+ α-syn+ Plasminogen;
Lane 3,6,9：Saline+ α-syn+ Plasminogen+EACA;
 Lane 4,7,10：Saline+ α-syn+ Plasminogen+aprotinin;

## Slide 4
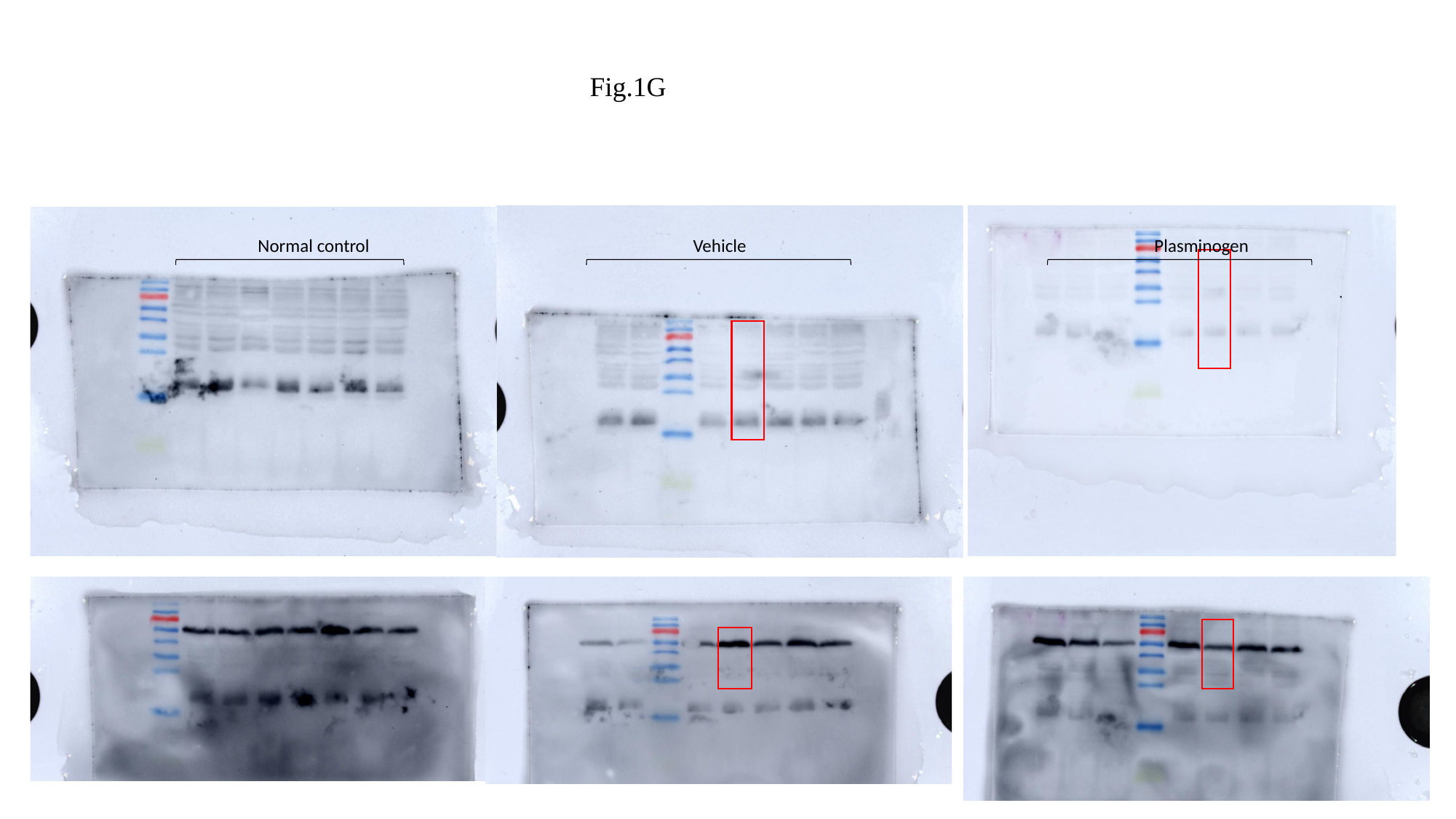

Fig.1G
Plasminogen
Normal control
Vehicle

## Slide 5
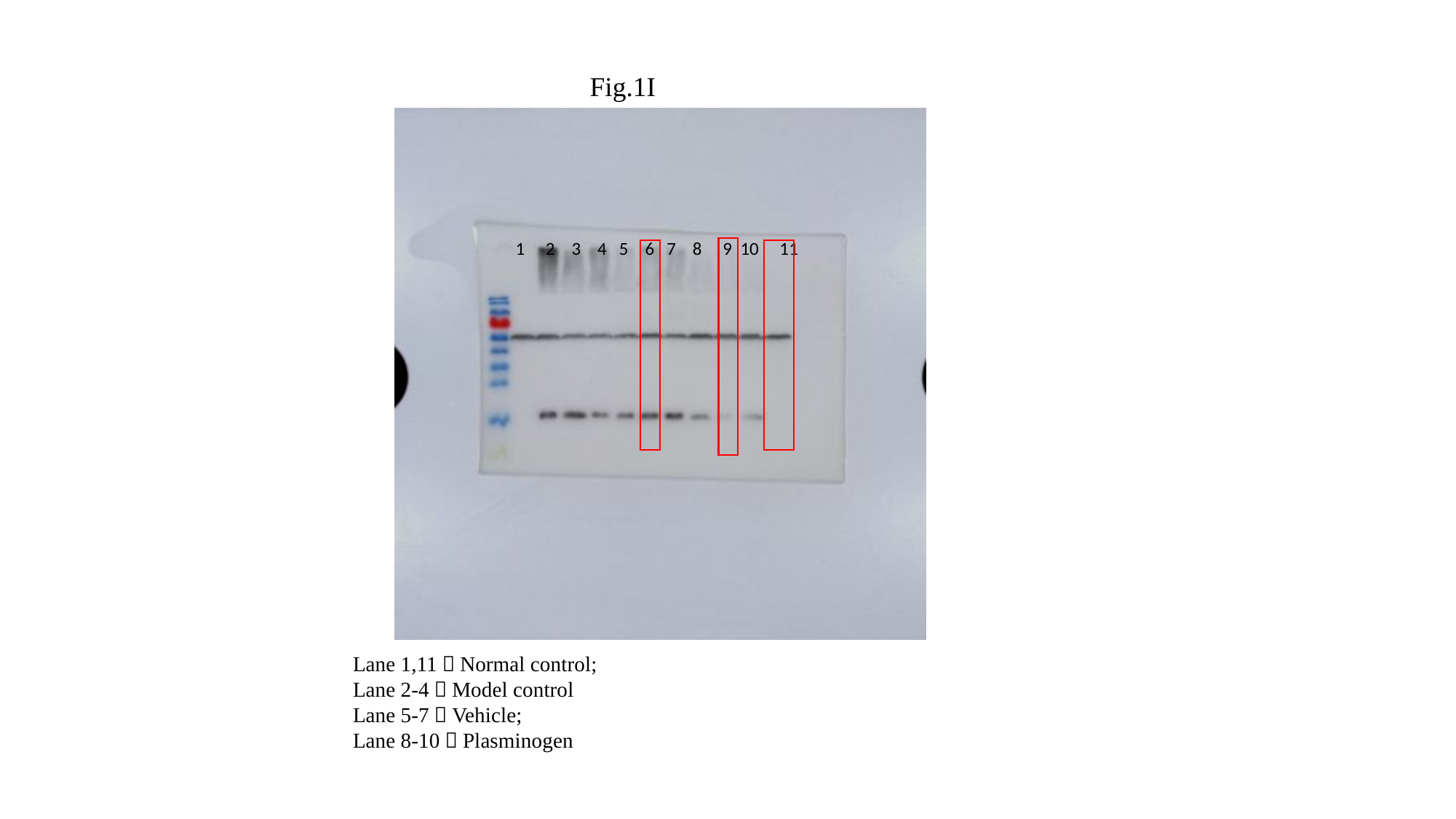

Fig.1I
1 2 3 4 5 6 7 8 9 10 11
Lane 1,11：Normal control;
Lane 2-4：Model control
Lane 5-7：Vehicle;
Lane 8-10：Plasminogen

## Slide 6
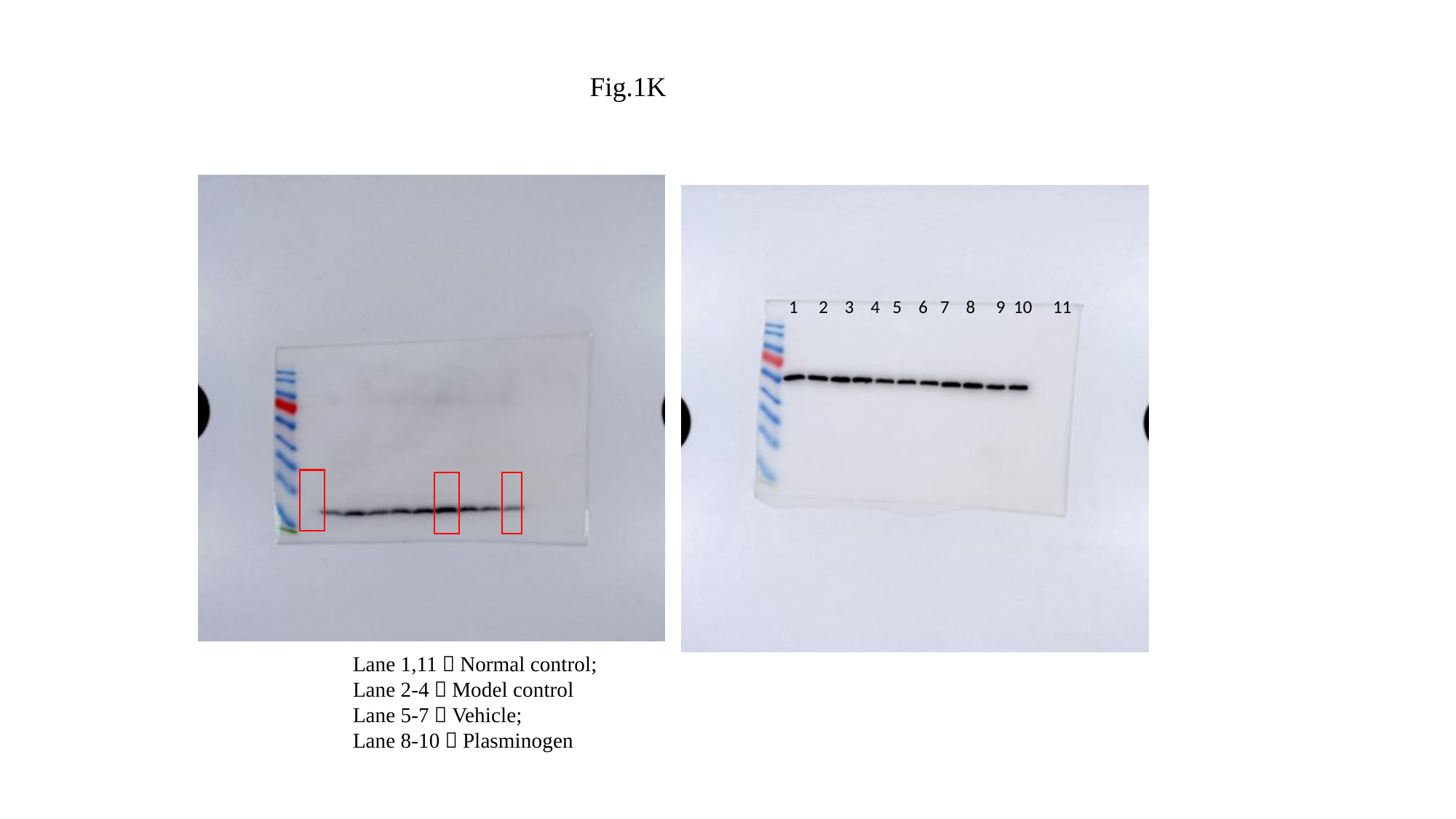

Fig.1K
1 2 3 4 5 6 7 8 9 10 11
Lane 1,11：Normal control;
Lane 2-4：Model control
Lane 5-7：Vehicle;
Lane 8-10：Plasminogen

## Slide 7
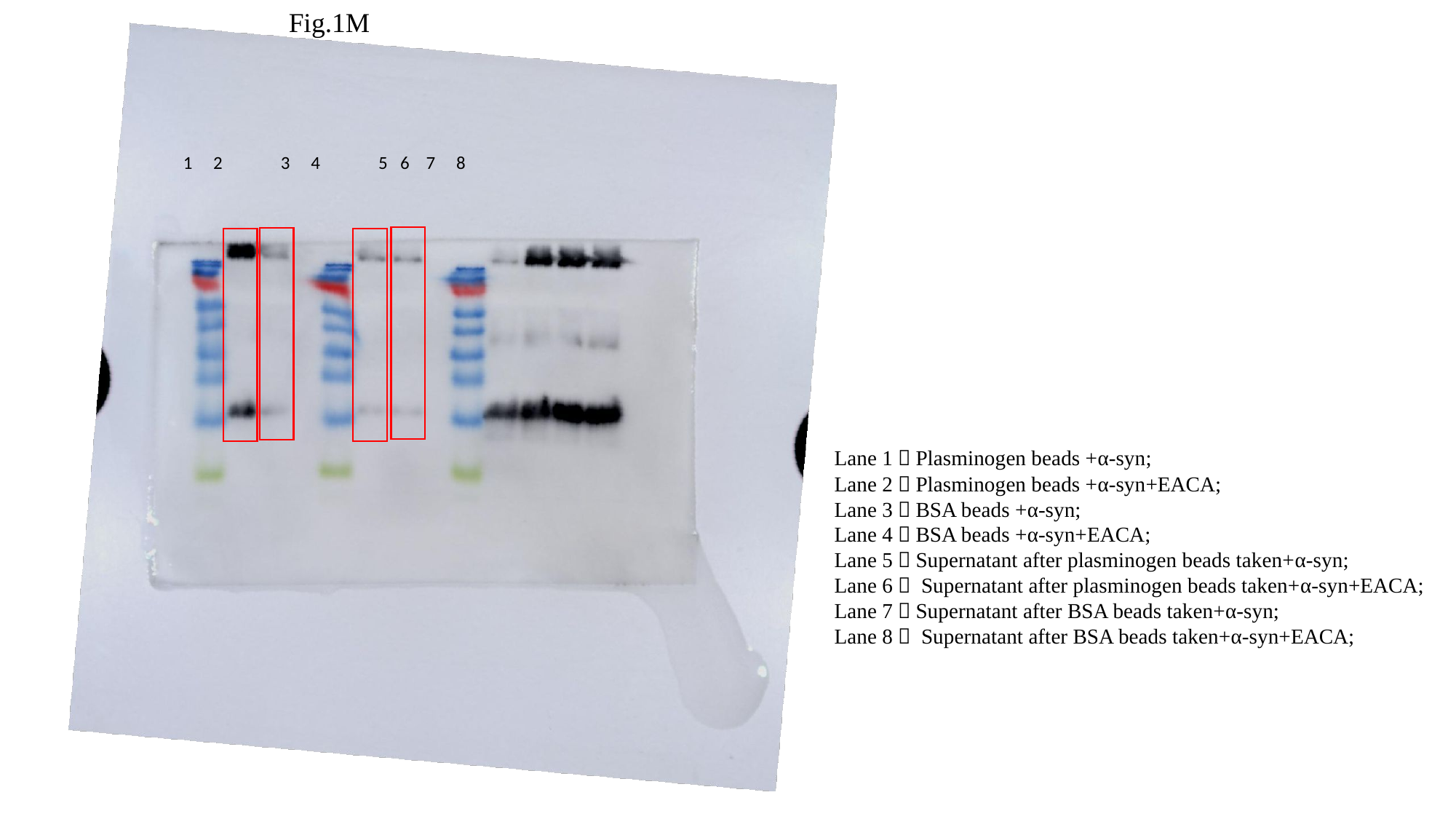

Fig.1M
1 2 3 4 5 6 7 8
Lane 1：Plasminogen beads +α-syn;
Lane 2：Plasminogen beads +α-syn+EACA;
Lane 3：BSA beads +α-syn;
Lane 4：BSA beads +α-syn+EACA;
Lane 5：Supernatant after plasminogen beads taken+α-syn;
Lane 6： Supernatant after plasminogen beads taken+α-syn+EACA;
Lane 7：Supernatant after BSA beads taken+α-syn;
Lane 8： Supernatant after BSA beads taken+α-syn+EACA;

## Slide 8
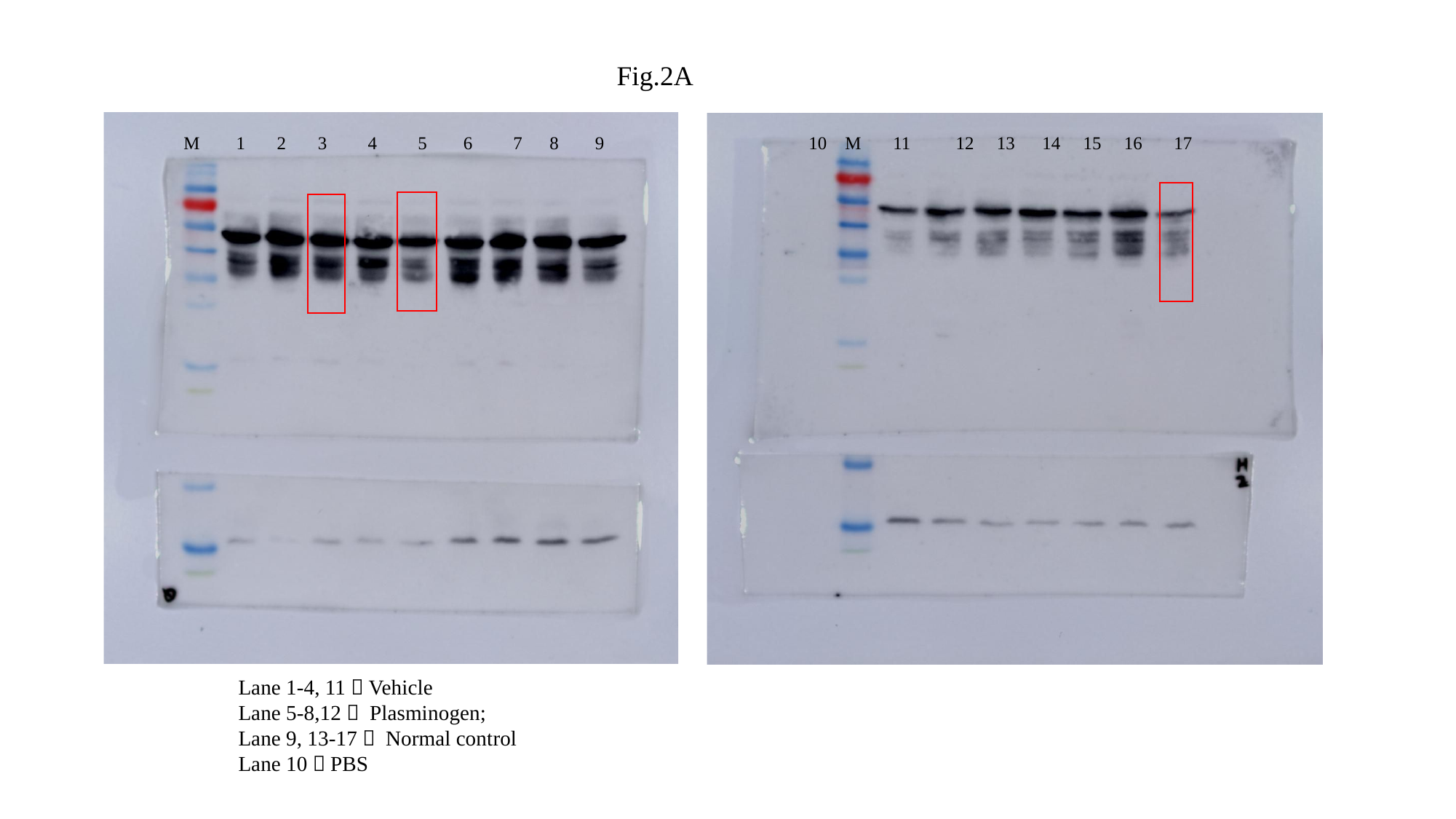

Fig.2A
M 1 2 3 4 5 6 7 8 9 10 M 11 12 13 14 15 16 17
Lane 1-4, 11：Vehicle
Lane 5-8,12： Plasminogen;
Lane 9, 13-17： Normal control
Lane 10：PBS

## Slide 9
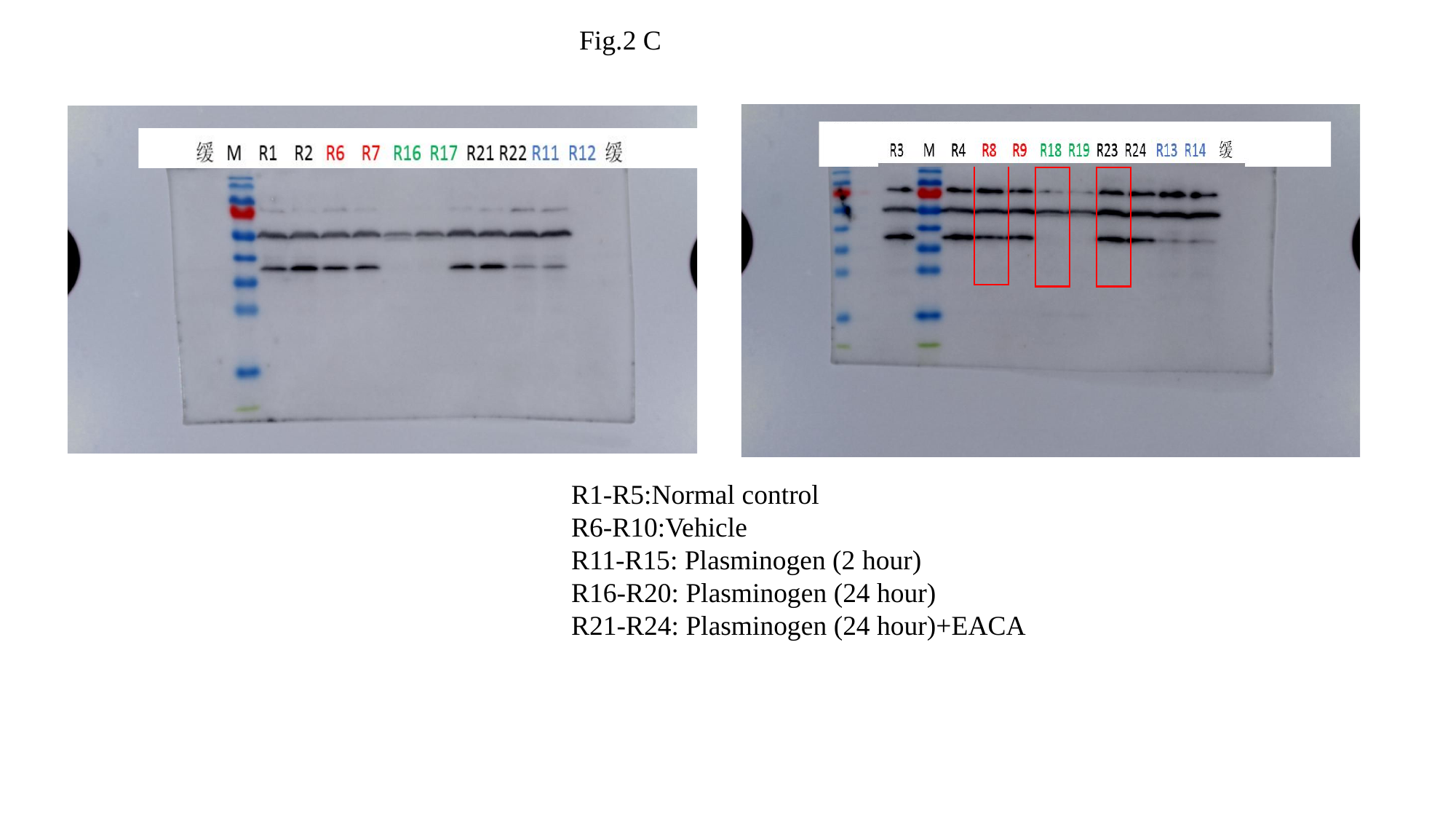

Fig.2 C
R1-R5:Normal control
R6-R10:Vehicle
R11-R15: Plasminogen (2 hour)
R16-R20: Plasminogen (24 hour)
R21-R24: Plasminogen (24 hour)+EACA

## Slide 10
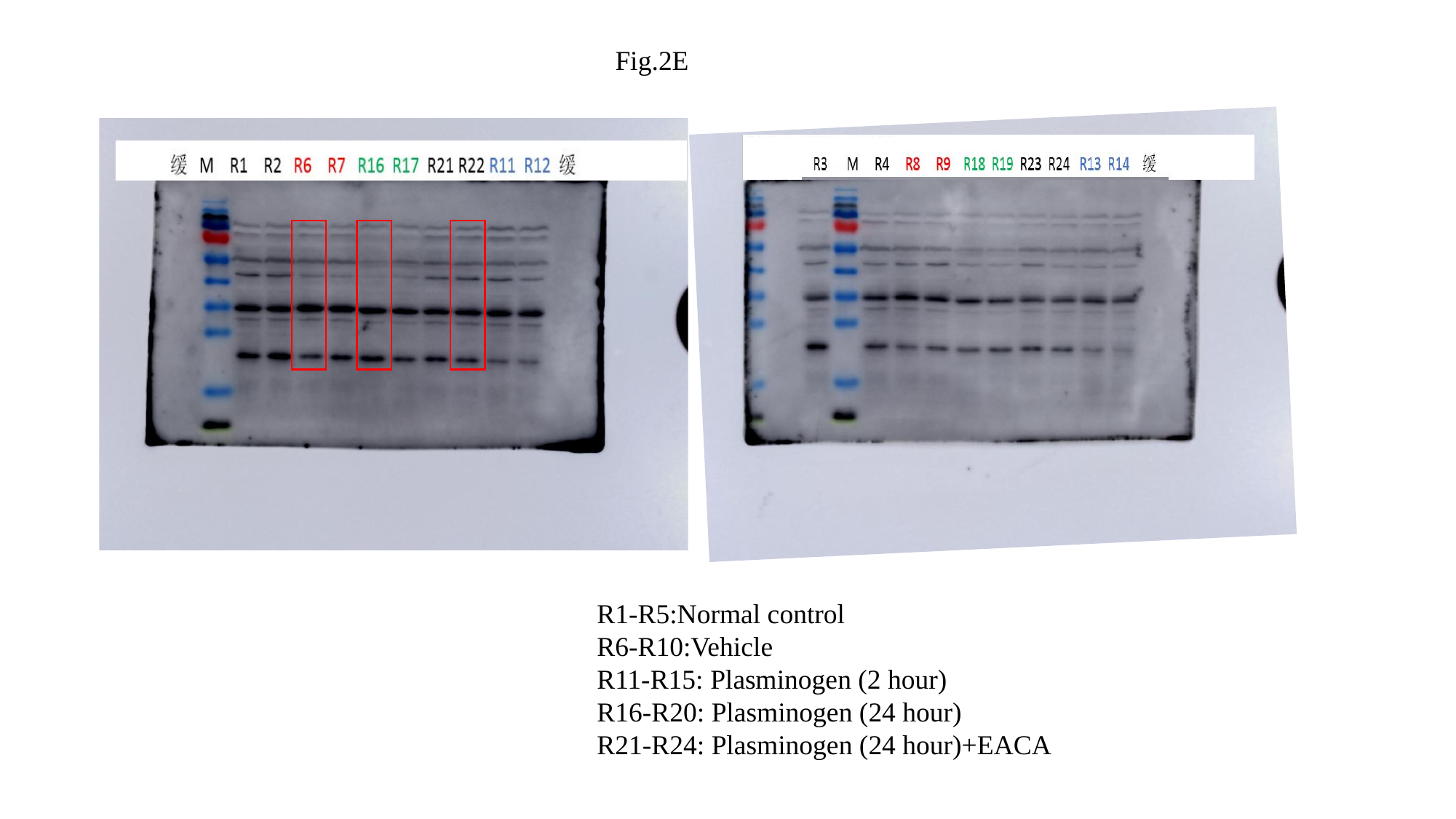

Fig.2E
R1-R5:Normal control
R6-R10:Vehicle
R11-R15: Plasminogen (2 hour)
R16-R20: Plasminogen (24 hour)
R21-R24: Plasminogen (24 hour)+EACA

## Slide 11
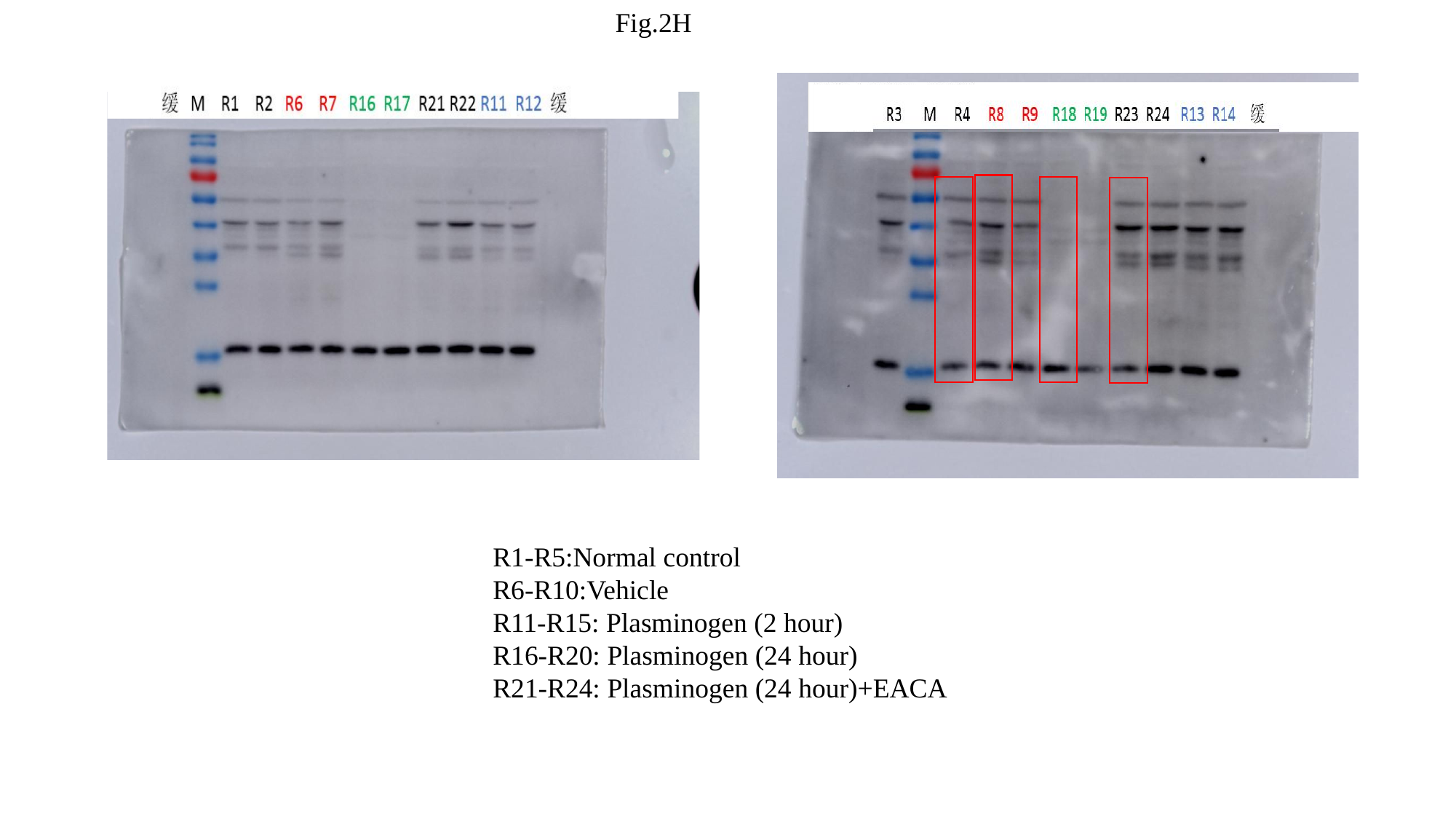

Fig.2H
R1-R5:Normal control
R6-R10:Vehicle
R11-R15: Plasminogen (2 hour)
R16-R20: Plasminogen (24 hour)
R21-R24: Plasminogen (24 hour)+EACA

## Slide 12
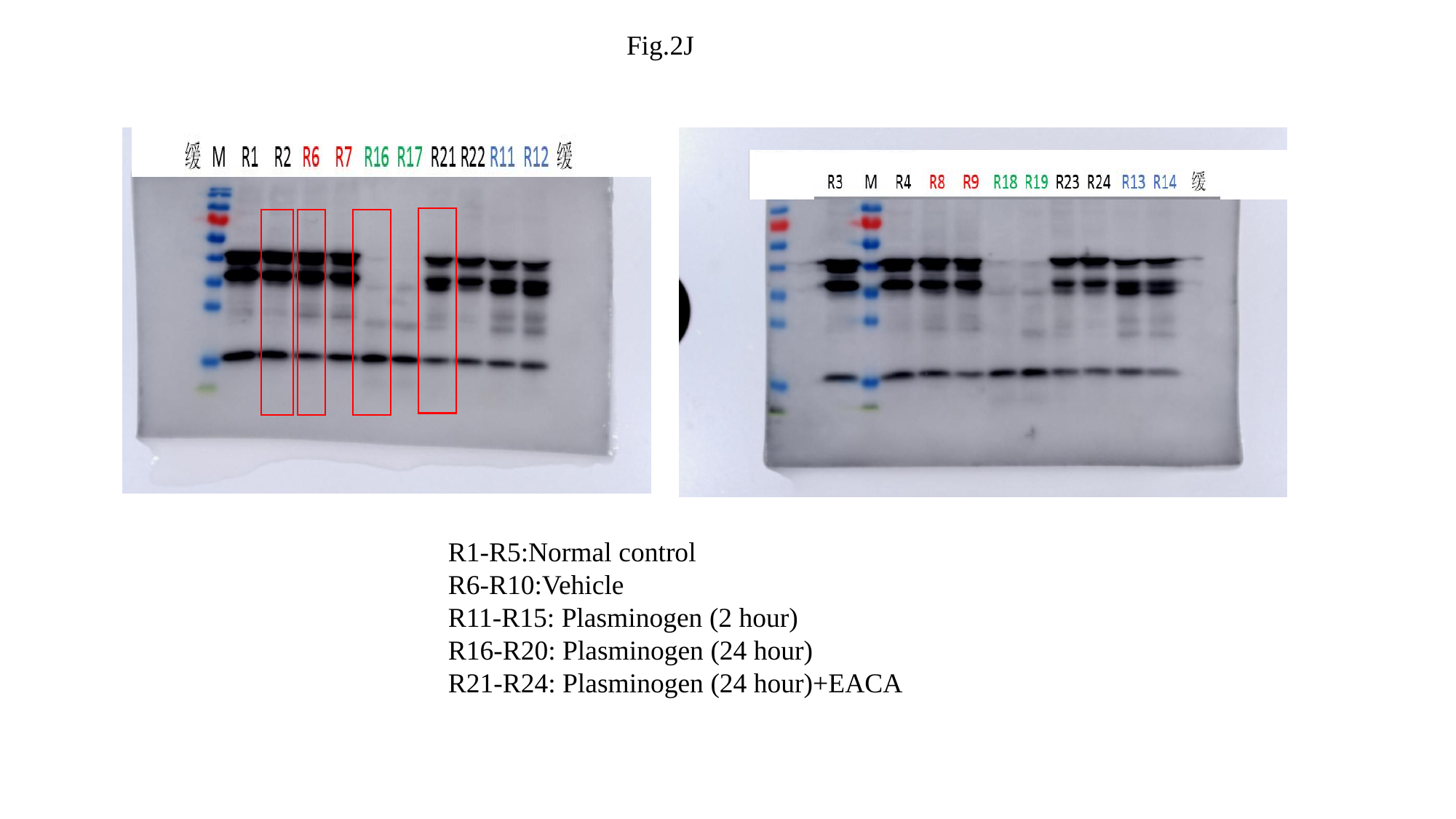

Fig.2J
R1-R5:Normal control
R6-R10:Vehicle
R11-R15: Plasminogen (2 hour)
R16-R20: Plasminogen (24 hour)
R21-R24: Plasminogen (24 hour)+EACA

## Slide 13
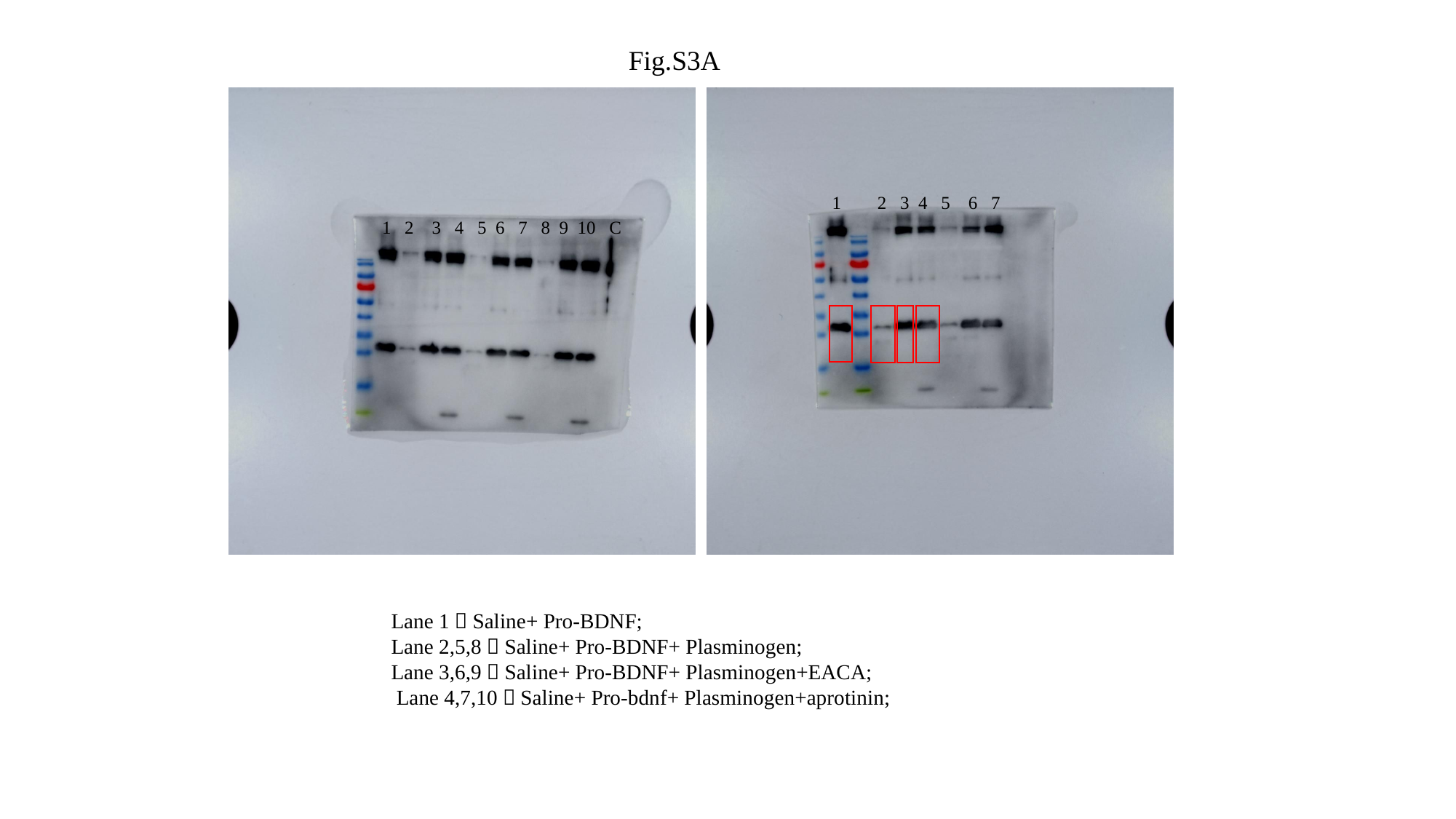

Fig.S3A
1 2 3 4 5 6 7
1 2 3 4 5 6 7 8 9 10 C
Lane 1：Saline+ Pro-BDNF;
Lane 2,5,8：Saline+ Pro-BDNF+ Plasminogen;
Lane 3,6,9：Saline+ Pro-BDNF+ Plasminogen+EACA;
 Lane 4,7,10：Saline+ Pro-bdnf+ Plasminogen+aprotinin;
