# Supplementary material for: Plasminogen degrades α-synuclein, Tau and TDP-43 and decreases dopaminergic neurodegeneration in mouse models of Parkinson’s disease
Source: Sci Rep. 2024 Apr 13;14:8581. doi: 10.1038/s41598-024-59090-8 (PMC11016066; doi:10.1038/s41598-024-59090-8)
Supplement: Supplementary file 2 — Supplementary Information 2. [file 41598_2024_59090_MOESM2_ESM.docx]

**Supplementary material**

**Table S1. List of the main reagents and antibodies used in the study**

| **Reagents/Antibodies** | **Company** | **Cat No.** |
| --- | --- | --- |
| 1-Methyl-4-phenyl-1,2,3,6-tetrahydropyridine (MPTP) | Sigma (China) | M0896 |
| Recombinant human α-syn protein | ChinaPeptides (Shanghai, China). | UniProtKB-P37840 |
| Recombinant human Pro-BDNF | Genscript (Nanjing, China) | UniProtKB-P23560 |
| Lipopolysaccharide (LPS) | Solarbio (China) | L8880 |
| Anti-α-syn antibody | Proteintech (China) | 10842-1-AP |
| Phospho-α-Synuclein (Ser129) (D1R1R) Rabbit mAb (P-S129 α-syn) | Cell Signaling Technology (China) | 23706S |
| Anti-Tau (phospho T231) antibody | Abcam (China) | ab151559 |
| Polyclonal anti-Tau (fusion protein Ag0354) antibody | Proteintech (China) | 10274-1-AP |
| Anti-tyrosine hydroxylase (TH) antibody | Proteintech (China) | 25859-1-AP |
| Anti-neurofilament (NF) antibody | Abcam (China) | ab207176 |
| Anti- dopamine transporter (DAT）antibody | Abcam (China) | ab7260 |
| Anti-ubiquitin antibody | Abcam (China) | ab7780 |
| Anti-LC3 antibody | Proteintech (China) | 18725-1-AP |
| Anti-TDP-43 antibody | Proteintech (China) | 12892-1-AP |
| XTSA520 | Alpha X Biotech (China) | AXT6202500 |
| XTSA620 | Alpha X Biotech (China) | AXT6502500 |
| DAPI | Boster (China) | 11K16B77 |
| HRP-conjugated anti-rabbit secondary antibody, made in goat | Abcam (China) | ab6721 |
| HRP Anti-Rabbit IgG (Peroxidase) Polymer Detection Kit, made in Goat | Vector laboratories (China) | MP7451 |
| Anti-BDNF antibody | Boster (China) | PB9075 |
| HRP conjugated anti-human plasminogen monoclonal antibody | Talengen Institute of Life Sciences | - |
| Substrate S-2251 | Chromogenix (China) | 82033239 |
| NSC34 cell | Otwo Biotech (China) | HTX1846 |
| Okadaic acid (OA) | Shanghai Yuanye Bio-Technology (China) | S30686-25 µg |
| Aminocaproic acid (EACA) | Sigma (China) | A2504-1 kg |
| Dynabeads M-280 Tosylactivated | Invitrogen (China) | 14203 |
| Bovine Serum Albumin (BSA) | Solarbio (China) | PC0001 |
| Dulbecco's modified eagle medium (DMEM) | Gibco (China) | 11965092 |
| Fetal bovine serum (FBS) | Every Green (China) | 11011-8611 |
| Dimethyl sulfoxide (DMSO) | Sigma (China) | D2438-5X10ML |
| Trypsin | Beyotime Biotechnology (China) | C0201-500 mL |
| Nuclear Protein Extraction Kit | Solarbio (China) | R0050 |
| RNA Easy Fast kit | Tiangen (China) | DP451 |
| Abstart One Step RT‒PCR Mix kit | Sangon Biotech (China) | B110025-0100 |
| Human plasminogen working standard | Talengen Institute of Life Sciences | - |
| Trifluoroacetic acid | Aladdin (China) | T103291 |
| Immobilon Western HRP Substrate | Millipore (China) | P36599 |
| Diaminobenzidine (DAB) | Vector laboratories (China) | SK-4100 |
| Aprotinin | Hangzhou Meiya Pharmaceutical Co., Ltd (China) | 160920-2 |
| Human plasminogen labeled with fluorescent dye of Alexa Fluor 488 | KMD bioscience (China) | - |

-: Indicates no information.

**Table S2: Primer information**

| **Gene** | **Sequence F** | **Sequence R** |
| --- | --- | --- |
| Α2-antiplasmin | TTCTCCTCAACGCCATCCA | GGTGAGGCTCGGGTCAAAC |
| PAI-1 | TCCAGCAGCTGAATTCCTG | GCTGGAGACATCTGCATCCT |
| tPA | CGAAAGCTGACGTGGGAATA | GTGTGAGGTGATGTCTGTGTAG |
| uPA | GCGCCTTGGTGGTGAAAAAC | GACACGCATACACCTCCGTT |
| Tubulin | TCGCCTAGATCACAAGTTTGATCT | ACCAACCTCCTCATAATCCTTC |

Fig. S1


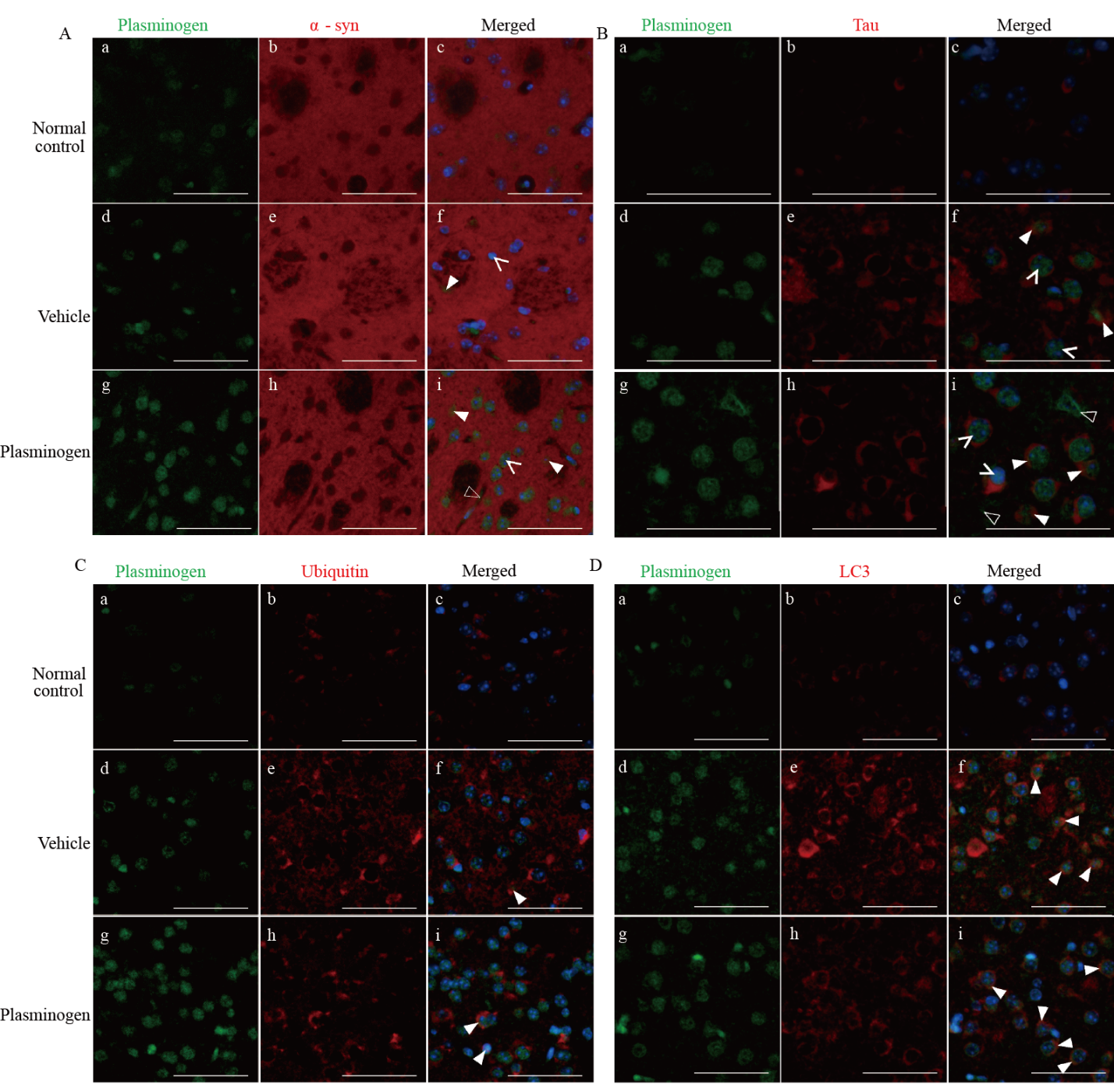


Fig. S1. Plasminogen colocalizes/is adjacent with α-syn and Tau and components of the ALP and UPS in striatum. A: Representative images showing co-immunofluorescence staining of plasminogen (green) and α-syn (red) and DAPI staining (nuclei, blue) in the striatum of PD model mice. B: Representative images showing co-immunofluorescence staining of plasminogen (green) and Tau (red) and DAPI staining (nuclei, blue) in the striatum of PD model mice. C: Representative images showing co-immunofluorescence staining of plasminogen (green) and ubiquitin (red) and DAPI staining (nuclei, blue) in the striatum of PD model mice. D: Representative images showing co-immunofluorescence staining of plasminogen (green) and LC3 (red) and DAPI staining (nuclei, blue) in the striatum of PD model mice. Normal control group: a-c; vehicle-treated group: d-f; plasminogen-treated group: g-i. Open arrow: localization of plasminogen in the nuclei, open triangle: localization of plasminogen in the cytoplasm, solid triangle: colocalization/adjacency of plasminogen and α-syn (Af, Ai), Tau (Bf, Bi), ubiquitin (Cf, Ci), or LC3 (Df, Di) in the cytoplasm; scale bar =50 μm.

Fig. S2


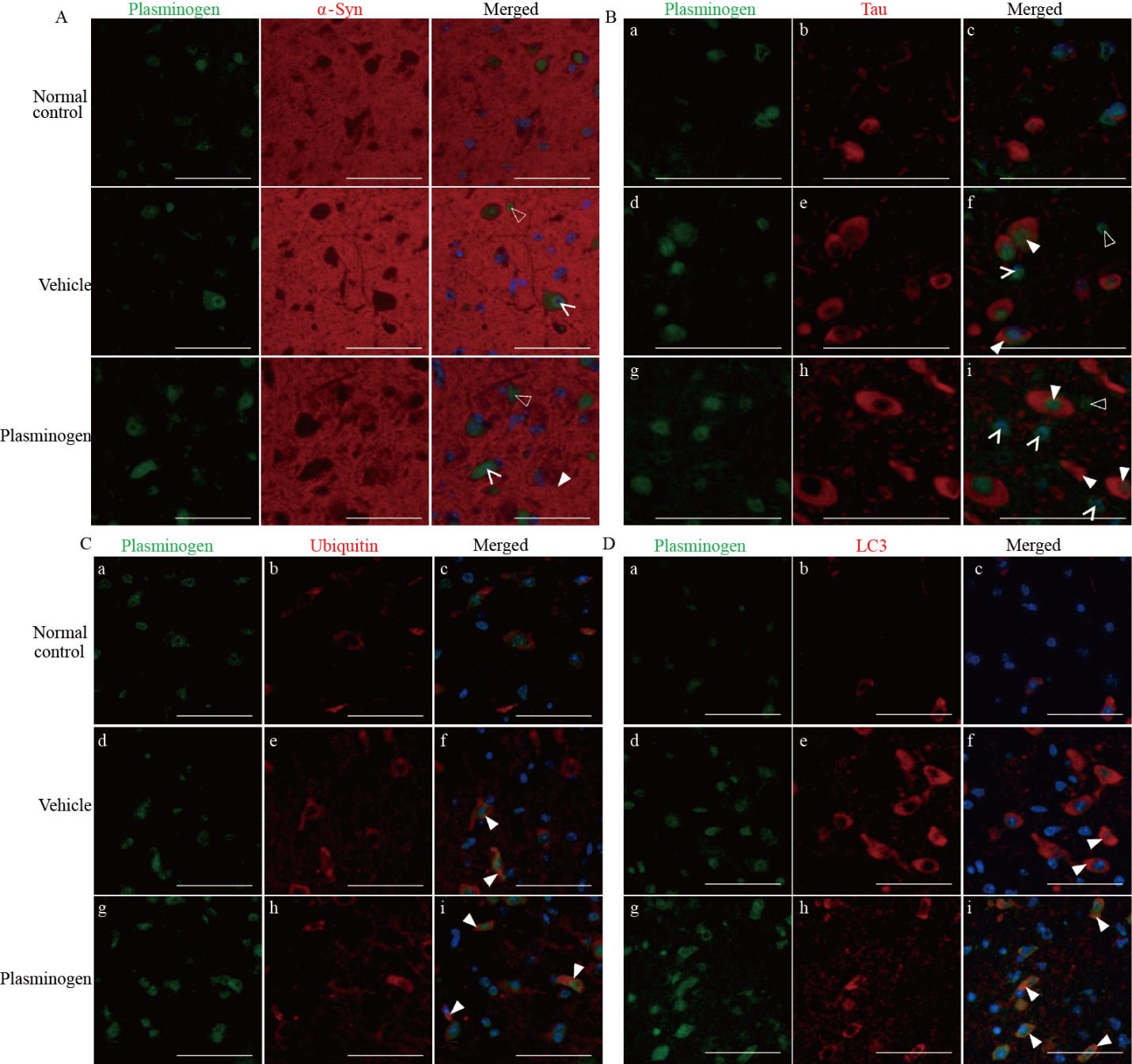


Fig. S2. Plasminogen colocalizes/is adjacent with α-syn and Tau and components of the ALP and UPS in substantia nigra. A: Representative images showing co-immunofluorescence staining of plasminogen (green) and α-syn (red) and DAPI staining (nuclei, blue) in the substantia nigra of PD model mice. B: Representative images showing co-immunofluorescence staining of plasminogen (green) and Tau (red) and DAPI staining (nuclei, blue) in the substantia nigra of PD model mice. C: Representative images showing co-immunofluorescence staining of plasminogen (green) and ubiquitin (red) and DAPI staining (nuclei, blue) in the substantia nigra of PD model mice. D: Representative images showing co-immunofluorescence staining of plasminogen (green) and LC3 (red) and DAPI staining (nuclei, blue) in the substantia nigra of PD model mice. Normal control group: a-c; vehicle-treated group: d-f; plasminogen-treated group: g-i. Open arrow: localization of plasminogen in the nuclei, open triangle: localization of plasminogen in the cytoplasm, solid triangle: colocalization/adjacency of plasminogen and α-syn (Af, Ai), Tau (Bf, Bi), ubiquitin (Cf, Ci), or LC3 (Df, Di) in the cytoplasm; scale bar =50 μm.

Fig. S3


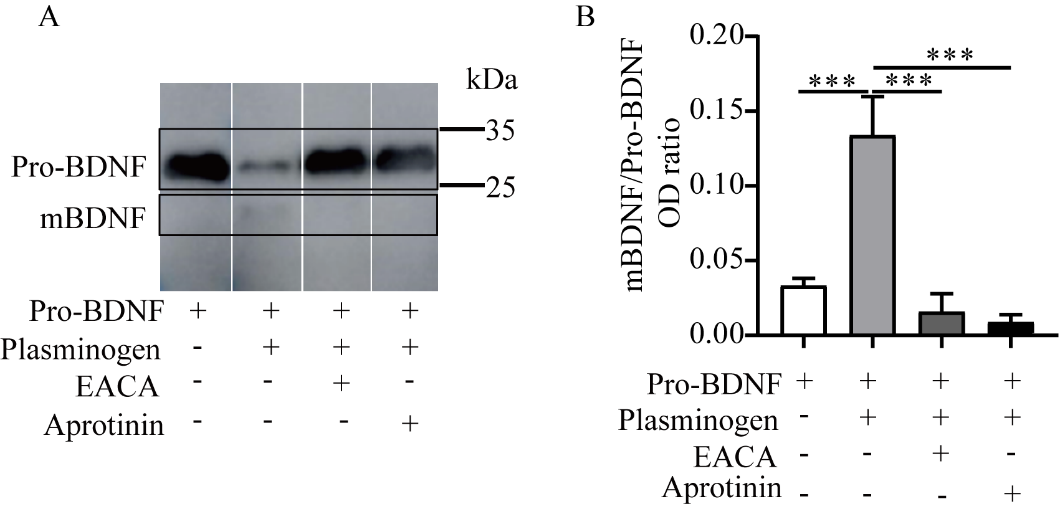


Fig. S3. Plasminogen promotes the activation and maturation of BDNF *in vitro*. Recombinant Pro-BDNF protein without incubation, or recombination Pro-BDNF protein that was incubated with plasminogen, plasminogen with EACA or plasminogen with aprotinin for 6 hours and further analyzed by WB. A: Representative WB showing Pro-BDNF (30-35 kDa) and mBDNF (18 kDa) levels after incubation; B: Quantitative analysis of the ratios of mBDNF/Pro-BDNF in Fig. S3A. n = 2 (for recombinant Pro-BDNF protein without incubation) or 5 (the rest groups) per group . ***, P < 0.001.
